# lncRNA *DRAIR* is downregulated in diabetic monocytes and modulates the inflammatory phenotype via epigenetic mechanisms

**DOI:** 10.1172/jci.insight.143289

**Published:** 2021-06-08

**Authors:** Marpadga A. Reddy, Vishnu Amaram, Sadhan Das, Vinay Singh Tanwar, Rituparna Ganguly, Mei Wang, Linda Lanting, Lingxiao Zhang, Maryam Abdollahi, Zhuo Chen, Xiwei Wu, Sridevi Devaraj, Rama Natarajan

**Affiliations:** 1 Department of Diabetes Complications and Metabolism, Arthur Riggs Diabetes and Metabolism Research Institute and Beckman Research Institute of City of Hope, Duarte, California, USA.; 2 Division of Pharmacology, CSIR-Central Drug Research Institute, Lucknow, India.; 3 Integrative Genomics Core, Beckman Research Institute of City of Hope, Duarte, California, USA.; 4 Baylor College of Medicine and Texas Children’s Hospital, Houston, Texas, USA.

**Keywords:** Inflammation, Metabolism, Diabetes, Monocytes, Noncoding RNAs

## Abstract

Long noncoding RNAs (lncRNAs) are increasingly implicated in the pathology of diabetic complications. Here, we examined the role of lncRNAs in monocyte dysfunction and inflammation associated with human type 2 diabetes mellitus (T2D). RNA sequencing analysis of CD14^+^ monocytes from patients with T2D versus healthy controls revealed downregulation of antiinflammatory and antiproliferative genes, along with several lncRNAs, including a potentially novel divergent lncRNA *diabetes regulated antiinflammatory RNA* (*DRAIR*) and its nearby gene *CPEB2*. High glucose and palmitic acid downregulated *DRAIR* in cultured CD14^+^ monocytes, whereas antiinflammatory cytokines and monocyte-to-macrophage differentiation upregulated *DRAIR* via KLF4 transcription factor. *DRAIR* overexpression increased antiinflammatory and macrophage differentiation genes but inhibited proinflammatory genes. Conversely, *DRAIR* knockdown attenuated antiinflammatory genes, promoted inflammatory responses, and inhibited phagocytosis. *DRAIR* regulated target gene expression through interaction with chromatin, as well as inhibition of the repressive epigenetic mark H3K9me2 and its corresponding methyltransferase G9a. Mouse orthologous *Drair* and *Cpeb2* were also downregulated in peritoneal macrophages from T2D *db/db* mice, and *Drair* knockdown in nondiabetic mice enhanced proinflammatory genes in macrophages. Thus, *DRAIR* modulates the inflammatory phenotype of monocytes/macrophages via epigenetic mechanisms, and its downregulation in T2D may promote chronic inflammation. Augmentation of endogenous lncRNAs like *DRAIR* could serve as novel antiinflammatory therapies for diabetic complications.

## Graphical abstract

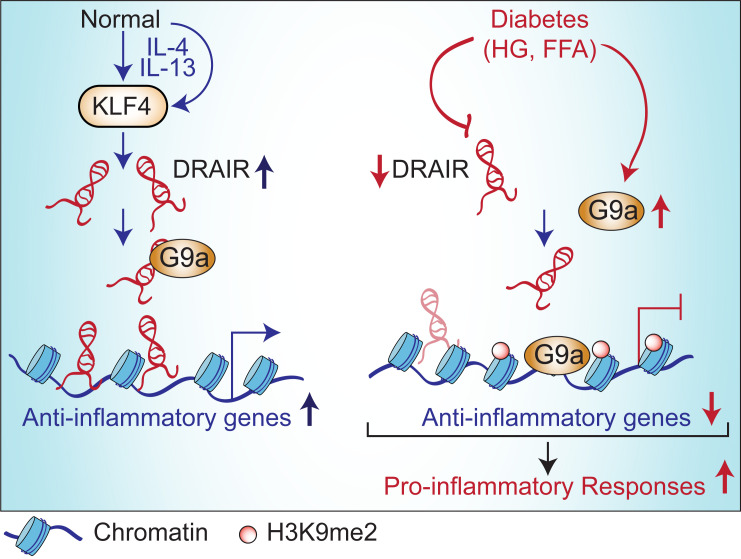

## Introduction

Systemic low-grade inflammation is a hallmark of type 2 diabetes mellitus (T2D) and contributes to the pathogenesis of several associated complications, including cardiovascular disease (1, 2). Activation of monocytes and macrophages plays an important role in inflammatory processes needed for protection against invading pathogens or toxins (3). Under physiological conditions, acute inflammation self-resolves via a balancing interplay between inflammatory and antiinflammatory mediators and is essential for tissue repair (4, 5). However, factors associated with inflammatory diseases like T2D, including high glucose (HG), elevated levels of advanced glycation end products (AGEs), and free fatty acids (e.g., palmitic acid [PA] and oxidized lipids), markedly enhance production of proinflammatory cytokines such as IL-1β, IL-6, and TNF-α and inhibit production of protective factors, leading to chronic inflammation. In addition, the diabetic milieu increases the number of monocytes (monocytosis), further increasing the burden for cardiovascular disease (5–10). Experimental studies have elucidated key molecular and signaling pathways involved in the activation of monocytes and macrophages. These include increased oxidative stress; production of ROS; and activation of various kinases, transcription factors (TFs) (e.g., NF-κB and STAT3), inflammasomes, and TLRs (5–7). Furthermore, epigenetic mechanisms have also been demonstrated in the activation of the proinflammatory phenotype of monocytes and macrophages (11). Evidence shows that blocking inflammatory signaling using antibodies against cytokines, cytokine receptor antagonists, lipid lowering drugs, and some antioxidants could reduce risk for metabolic disease and vascular complications (12–15). However, these therapeutic strategies are not always fully effective in preventing progression and recurrence of cardiometabolic disease. Thus, further understanding of the precise molecular mechanisms associated with chronic inflammation is needed to develop much-needed new and effective therapies.

Noncoding RNAs (ncRNAs) like microRNAs (miRNAs) and long noncoding RNAs (lncRNAs) have emerged as key regulators of gene expression mediating functions of monocytes and macrophages such as inflammation, innate immunity, differential response to endotoxemia, cholesterol homeostasis, and macrophage polarization (16, 17). lncRNAs are defined as > 200 nucleotide–long transcripts with no coding potential. They regulate gene transcription via diverse mechanisms depending on their subcellular localization. Nuclear lncRNAs can regulate gene expression by interacting with chromatin, or they can act as a scaffolds, decoys, or recruiters of chromatin modifying factors/complexes and TFs to alter epigenetic states at target gene promoters and enhancers. On the other hand, cytoplasmic lncRNAs can regulate functions of translation factors, signaling proteins, and miRNAs (16, 17).

Emerging evidence shows that lncRNAs can play functional roles in diabetic vascular disease (18–20). Our recent studies demonstrated the involvement of 3 lncRNAs in the regulation of the monocyte/macrophage inflammatory phenotype in diabetes and metabolic syndrome in mice and humans. Two of these lncRNAs, *E330013P06* and *Dnm3os*, were upregulated in macrophages from T2D *db/db* mice and T2D humans relative to healthy nondiabetic controls, and these lncRNAs had proinflammatory properties (21, 22). In contrast, another lncRNA, *Mist*, was downregulated in macrophages from high-fat diet–induced obese mice, as well as in adipose tissue macrophages from humans with obesity and metabolic syndrome, and exhibited antiinflammatory properties (23). Further mechanistic studies demonstrated that disruption of lncRNA-regulated epigenetic mechanisms under diabetic or obese conditions can facilitate proinflammatory phenotypes in macrophages (22, 23). Several lncRNAs dysregulated in cardiometabolic disease were also identified in human monocytes (24). However, very little is known about lncRNAs in human monocytes with protective effects that might assist in resolution of inflammation in T2D. Such knowledge could assist in the development of newer therapies for diabetes and its associated chronic inflammatory disorders.

In this study, we compared the transcriptome in CD14^+^ monocytes from T2D volunteers versus monocytes from nondiabetic volunteers to gain insights into the role of differentially expressed lncRNAs in monocyte dysfunction. We found that not only many inflammatory genes were upregulated, but several antiinflammatory and antiproliferative genes were downregulated; furthermore, several lncRNAs were dysregulated in T2D monocytes versus controls. Among the differentially expressed lncRNAs, we selected 1 potentially novel lncRNA for further characterization, which we named *diabetes regulated antiinflammatory lncRNA* (*DRAIR*). *DRAIR* expression was downregulated in T2D monocytes and in cultured human monocytes treated with HG and PA, but it was upregulated by antiinflammatory cytokines IL-4 and IL-13. Functional and mechanistic studies demonstrated that *DRAIR* increases the expression of antiinflammatory genes via interaction with chromatin and modulation of repressive epigenetic histone modifications at target gene promoters. Furthermore, the antiinflammatory function of *Drair* (mouse ortholog) was also observed in vivo in mice. Together, these studies demonstrate that lncRNA *DRAIR* regulates the antiinflammatory phenotype via epigenetic mechanisms in monocytes and that its downregulation in diabetes promotes chronic inflammation.

## Results

### T2D is associated with reduced expression of antiinflammatory and antiproliferation genes in human CD14^+^ monocytes.

**Figure 1:**
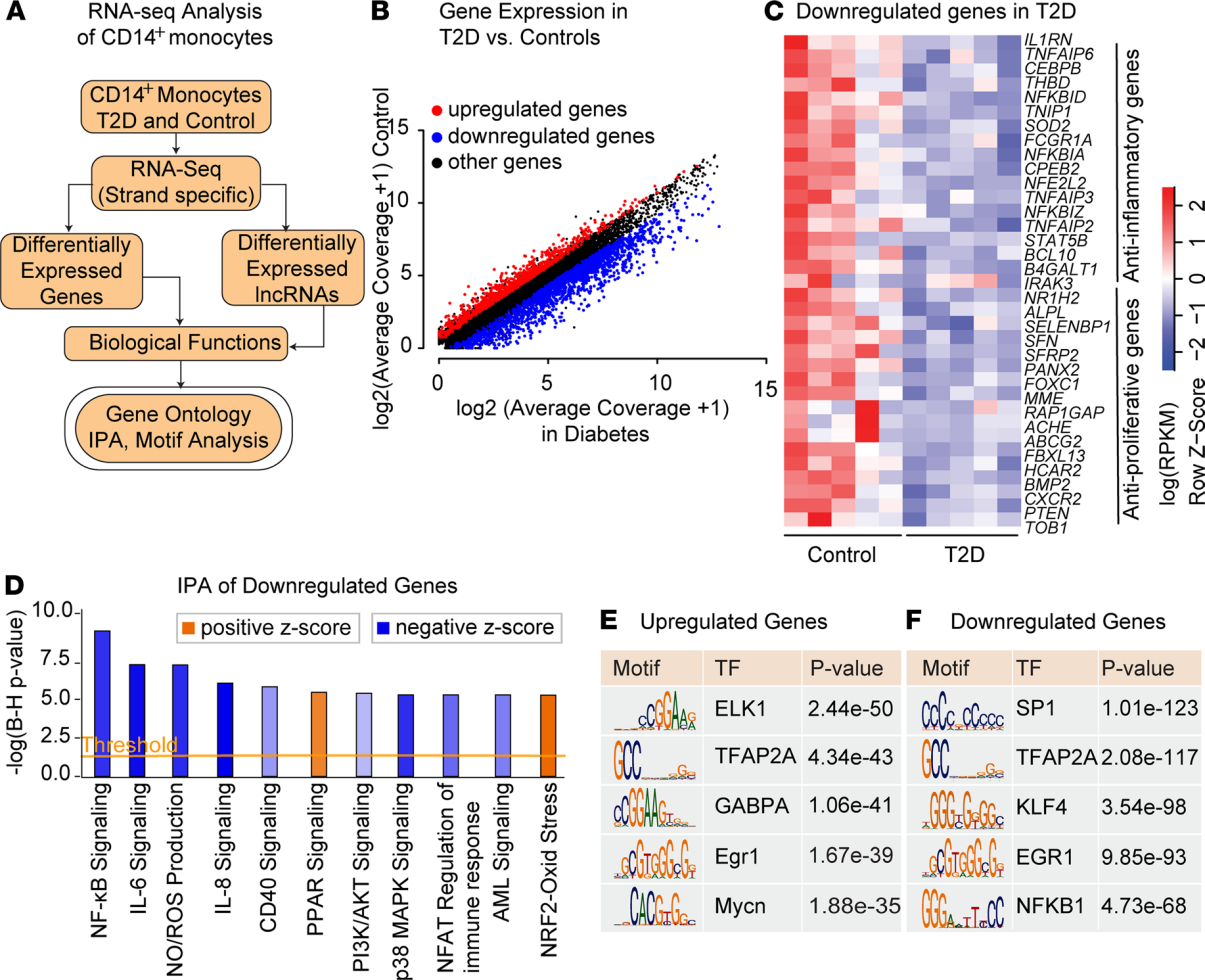
Type 2 diabetes inhibits antiinflammatory and antiproliferation genes in human CD14^+^ monocytes. (**A**) Scheme showing RNA-seq analysis pipeline and downstream analyses of CD14^+^ monocytes from volunteers with and without T2D. (**B**) Scatter plot of differentially expressed genes (DEGs) in T2D monocytes versus controls (log_2_ fold change ≥ 2, FDR < 0.05 versus control monocytes, *n* = 5 each). (**C**) Heatmap showing downregulation of key antiinflammatory and antiproliferative genes in T2D monocytes. (**D**) Enrichment of canonical signaling pathways associated with inflammation in downregulated genes. B–H, Benjamini-Hochberg. (**E** and **F**) Transcription factor (TF) motifs enriched in the promoters (–1000 bp to +500 bp) of DEGs in T2D monocytes. Adjusted *P* values (B–H method) are shown.

Although evidence shows that T2D promotes monocyte activation and monocytosis associated with chronic inflammation, the dysregulated gene expression and the regulatory role of lncRNAs in these inflammatory processes are unclear. To examine this further, we performed strand-specific RNA sequencing (RNA-seq) analysis to profile changes in the transcriptome of CD14^+^ monocytes obtained from human volunteers with T2D (T2D monocytes) relative to control healthy volunteers without diabetes (control monocytes) (Figure 1A and Supplemental Table 1; supplemental material available online with this article; https://doi.org/10.1172/jci.insight.143289DS1). T2D monocytes exhibited extensively altered transcriptomes with upregulation of 993 genes and downregulation of 1865 genes (fold-change ≥ 2, average coverage ≥ 1, FDR ≤ 0.05, *n* = 5 per group) versus control monocytes (Figure 1B). Several inflammatory genes were upregulated (Supplemental Figure 1A), in line with previous studies (25–27). Interestingly, there was much greater reduction in the expression of several key antiinflammatory, antioxidant, and antiproliferative genes, including *IL1RN* that codes for IL-1 receptor antagonist (IL-1Ra) and *SOD2* and *PTEN* — in T2D monocytes, respectively (Figure 1C) — suggesting that T2D is associated with the loss of protective genes.

Gene ontology (GO) analysis showed enrichment of processes associated with translation, wound healing, ncRNA processing, and immune cell activation in the upregulated genes. Downregulated genes showed enrichment of intracellular signaling, immune response, and apoptosis (Supplemental Figure 1, B and C). Ingenuity Pathway Analysis (IPA) revealed enrichment of inflammatory response under the Diseases and Functions category in both the upregulated and downregulated genes (Supplemental Figure 1, D and E). Moreover, IPA also revealed enrichment of canonical pathways related to fatty acid oxidation and nitric oxide (NO) signaling among upregulated genes (Supplemental Figure 1F). Downregulated genes were enriched with several overlapping networks including NF-κB signaling, PI3K/Akt activation, nitric oxide (NO)/ROS production, and the IL-6 pathway (Figure 1D and Supplemental Figure 1G). Together, these data suggest that disruption of protective mechanisms/factors may activate inflammatory pathways in T2D. Motif analysis revealed that promoters of differentially expressed genes (DEGs) were enriched in binding sites for key TFs such as NF-κB, Egr1, and KLF4 (Figure 1, E and F), which are known to be involved in inflammation and macrophage polarization (28). These results suggest that T2D-induced changes in the monocyte transcriptome can dysregulate genes associated with key monocyte/macrophage functions — including inflammatory phenotype, apoptosis, proliferation, and phagocytosis — that can contribute to inflammatory diabetes complications, including cardiovascular disease.

### Dysregulation of lncRNAs in T2D monocytes, including a potentially novel lncRNA DRAIR.

**Figure 2:**
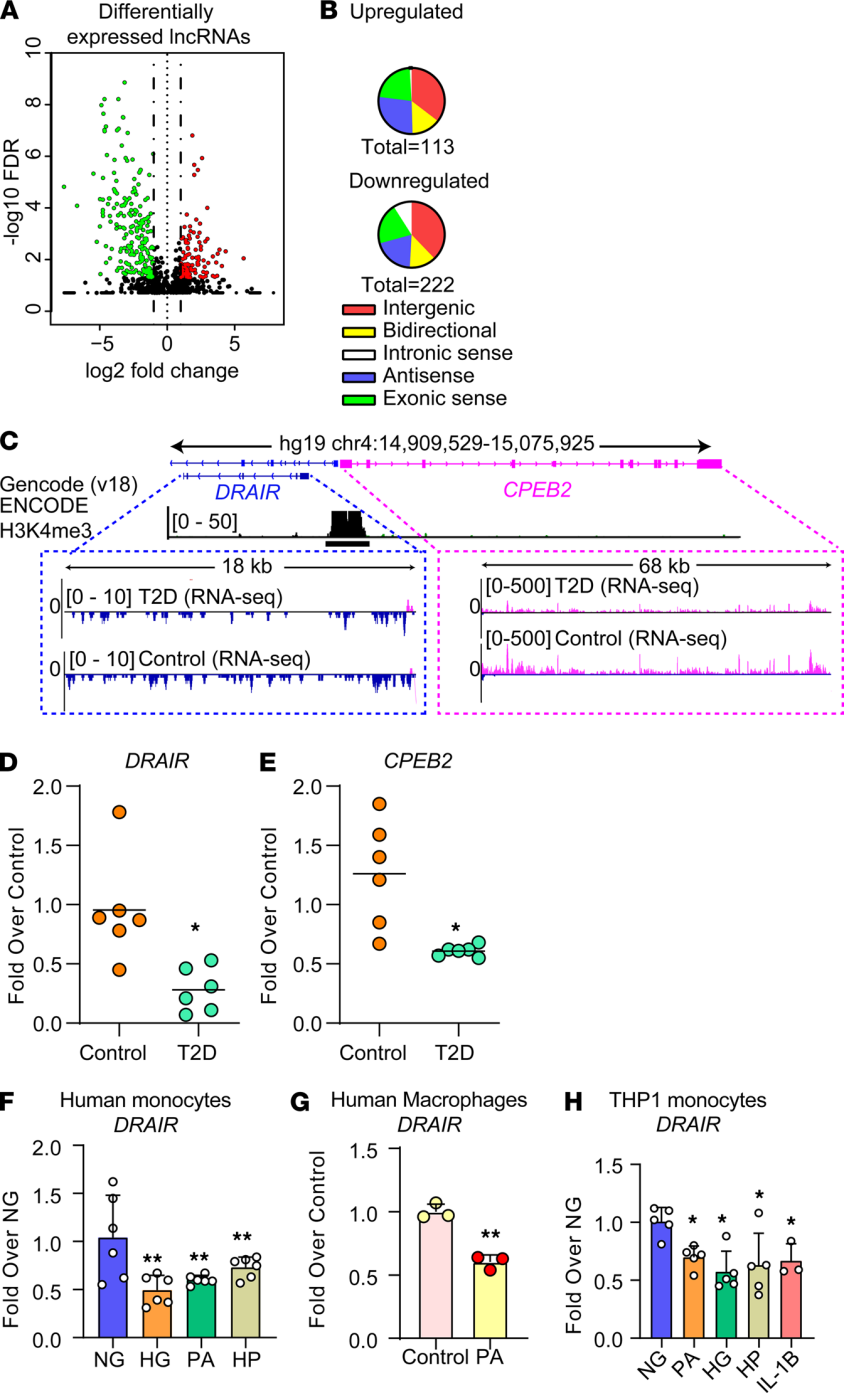
Dysregulation of lncRNA *DRAIR* in type 2 diabetes. (**A** and **B**) Volcano plot of differentially expressed lncRNAs in T2D monocytes versus controls (**A**) and their genomic classification (**B**). (**C**) Schematic showing genomic organization of lncRNA *DRAIR* and nearby *CPEB2* gene, along with RNA-seq tracks from CD14^+^ monocytes from T2D and control subjects. *DRAIR* and *CPEB2* show downregulation in T2D monocytes versus controls (log_2_ fold = –1.75, FDR = 0.029, and log_2_ fold = –2.19, FDR = 1.18 x 10^–5^, respectively; *n* = 5 each). H3K4me3 track from CD14^+^ monocytes (from ENCODE) is shown to indicate shared promoter region for both genes. Map not drawn to scale. (**D** and **E**) qPCR validation of *DRAIR* and *CPEB2* downregulation in T2D monocytes versus control (*P < 0.05, by *t* tests, *n* = 6). (**F**–**H**) qPCR results showing downregulation of *DRAIR* in primary human CD14^+^ monocytes (**F**), primary human macrophages (**G**), and THP1 monocytes (**H**) treated with normal glucose (NG, 5.5 mM), high glucose (HG, 25 mM, 72 hours), palmitic acid (PA, 200 μM, 24 hours), and HG + PA (HP) and IL-1β (10 ng/mL). *P < 0.05 and **P < 0.01, as determined by 1-way ANOVA and Dunnett’s multiple-comparison test in **F** and **H** (*n* = 3–6) and *t* test in **G** (*n* = 3).

To elucidate the role of lncRNAs in T2D-induced monocyte dysfunction, we further analyzed differentially expressed lncRNAs by assessing potential open reading frames, as described (21). We found that 335 lncRNAs were differentially expressed in T2D monocytes (Figure 2A), including those expressed from bidirectional promoters and designated as divergent transcripts (Figure 2B). GO analyses of nearby (±250 kb) DEGs revealed enrichment of inflammation and immune cell functions near downregulated lncRNAs (Supplemental Figure 2A), while metabolic processes were enriched in DEGs near upregulated lncRNAs (Supplemental Figure 2B).

Next, we tested our hypothesis that key lncRNAs downregulated in T2D may alter the inflammatory phenotype of monocytes and macrophages. We focused on a differentially expressed lncRNA annotated as *CPEB2-AS* (hg19), whose regulation and function have not been previously studied. *CPEB2-AS* is divergently transcribed adjacent to the cytoplasmic element binding protein 2 (*CPEB2*) gene on human chromosome 4. Both share the same promoter as evidenced by H3K4me3 enrichment (Figure 2C). Divergent transcripts have been reported in development (29), but their function in T2D-induced inflammation is unknown. Moreover, the nearby *CPEB2* belongs to the CPEB family, which has reported antiinflammatory functions (30), suggesting that *CPEB2-AS* may regulate inflammatory pathways. Based on subsequent functional assessment studies, we renamed *CPEB2-AS* as *DRAIR*.

RNA-seq data revealed that the *DRAIR* and nearby *CPEB2* were significantly downregulated in monocytes from T2D patients (Figure 2C). Using reverse transcription followed by quantitative PCR (qPCR), we validated the significant downregulation of *DRAIR* and *CPEB2* in CD14^+^ monocytes from T2D patients versus controls (Figure 2, D and E). T2D is associated with elevated blood glucose and circulating levels of free fatty acids such as PA. Therefore, we examined the effect of HG and PA in CD14^+^ monocytes from healthy volunteers in vitro. Monocytes were cultured in normal glucose (NG, 5.5 mM) or HG (25 mM) for 3 days. In the last 24 hours, NG and HG cells were also treated with PA (200 μM), referred to as PA and HG + PA (HP) groups, respectively. qPCR analysis showed that — relative to NG — PA, HG, or HP significantly inhibited *DRAIR* expression (Figure 2F). PA also downregulated *DRAIR* in CD14^+^ monocytes converted to macrophages (Figure 2G). Furthermore, HG, PA, and an inflammatory cytokine IL-1β (10 ng/mL, 24 hours), whose levels are elevated in T2D (2) also downregulated *DRAIR* expression in the human THP1 monocytic cell line (Figure 2H). These results demonstrate that lncRNA *DRAIR* is downregulated in T2D and by major pathological factors elevated in T2D, suggesting that it may regulate antiinflammatory processes in monocytes.

### Characterization of DRAIR and its subcellular localization.

Bioinformatics analysis using PhyloCSF and Coding Potential Calculator 2 suggested that *DRAIR* lacks coding potential (Supplemental Figure 3A). This was further confirmed experimentally in an in vitro–coupled transcription translation system using *DRAIR* cDNA cloned into the pcDNA3.1 expression plasmid as a template that showed absence of protein products (Supplemental Figure 3, B and D) similar to no template control (NTC) reactions. The positive control luciferase (LUC) transcript expressed a 62 kDa protein, as expected.

Because lncRNA functions are dependent upon subcellular localization, we next examined *DRAIR* levels in the cytoplasmic and nuclear fractions isolated from THP1 monocytes and THP1 cells differentiated into macrophages using phorbol myristate acetate (PMA, 20 ng/mL). qPCR showed that *DRAIR* levels were highly enriched in nuclear fractions from THP1 monocytes and macrophages. As expected, the known nuclear lncRNA *NEAT1* and coding RNA *PPIA* showed enrichment in nuclear and cytosolic fractions, respectively (Supplemental Figure 4, A–F). Nuclear localization of *DRAIR* was further confirmed by RNA-fluorescence in situ hybridization assays in THP1 macrophages using fluorescently labeled *DRAIR* probes (Supplemental Figure 4G). Furthermore, *DRAIR* was also found to be enriched in chromatin fractions versus soluble nuclear extracts similar to *NEAT1*, a known chromatin-associated lncRNA (Supplemental Figure 4, H and I), suggesting nuclear localized *DRAIR* might have functions related to transcriptional regulation in monocyte/macrophages.

### DRAIR downregulates the inflammatory phenotype in monocytes and macrophages.

**Figure 3:**
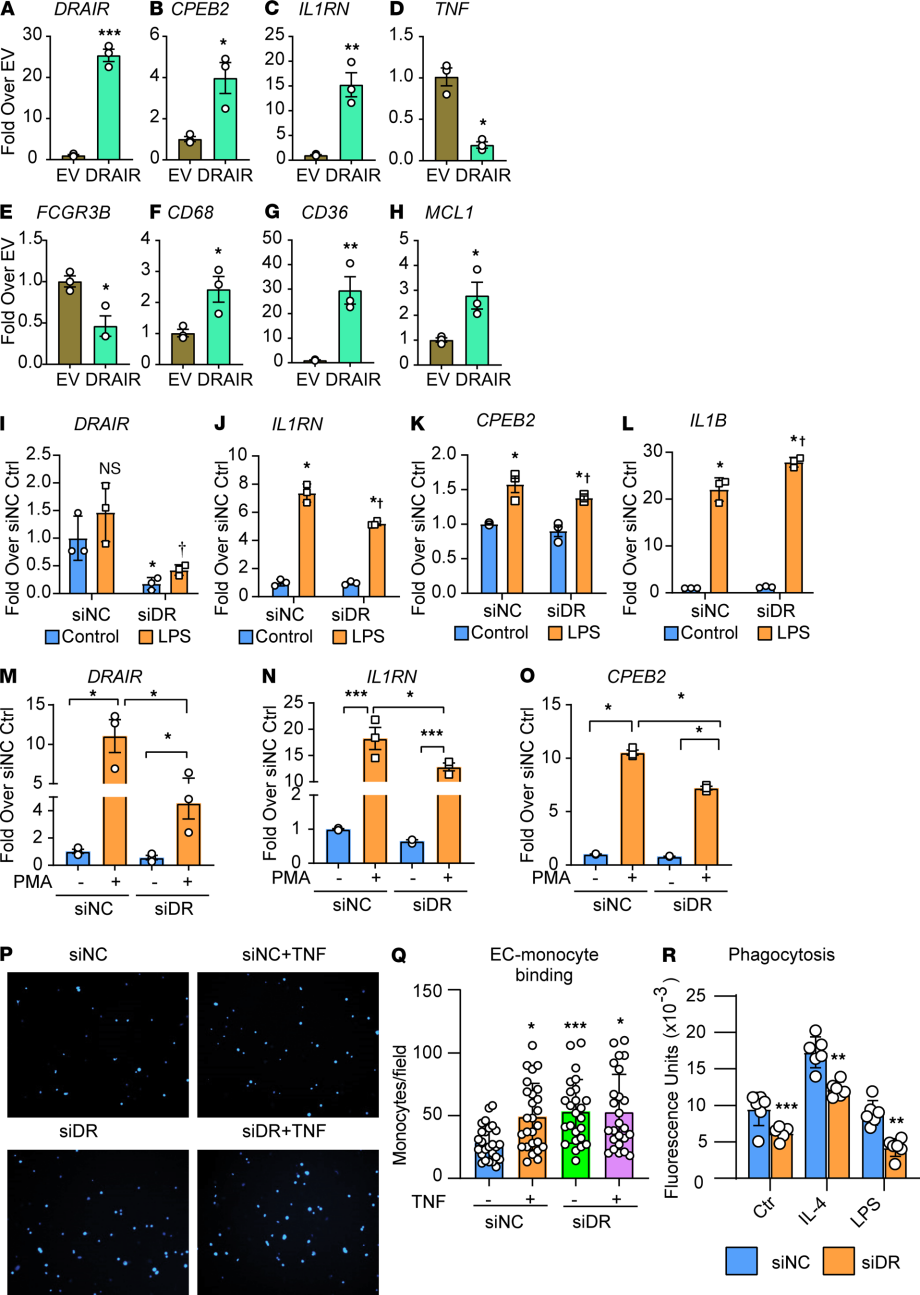
Effect of *DRAIR* overexpression or knockdown on proinflammatory gene expression and phenotype of THP1 monocytes. (**A**–**H**) Effects of *DRAIR* overexpression. qPCR analysis of indicated genes in THP1 cells transduced with lentivruses expressing *DRAIR* or a control vector (EV). *P < 0.05; **P < 0.01; ***P < 0.001, by *t* test (*n* = 3). (**I**–**L**) Effects of *DRAIR* knockdown with siRNAs. THP1 cells were transfected with control siRNA (siNC) or siRNA targeting *DRAIR* (siDR). Two days later, cells were treated with or without with LPS (100 ng/mL) for 24 hours, and gene expression was analyzed by qPCR. Results expressed as fold over siNC control. (**M**–**O**) qPCR analysis of THP1 cells transfected with siNC or siDR treated ± PMA (20 ng/mL) for 24 hours. Results expressed as fold over siNC control (Ctrl). For **I**–**O**, *P < 0.05; ***P < 0.001 versus siNC control and †*P* < 0.05 versus siNC LPS as determined by 2-way ANOVA (I–L), 1-way ANOVA (M–O), and Sidak’s multiple-comparison test (*n* = 3). (**P** and **Q**) THP1 cells transfected with siDR or siNC were treated ± TNF-α (10 ng/mL, 3 hours), labeled with DAPI, and used in monocyte–endothelial cell (monocyte-EC) adhesion assays. Images of bound monocytes were collected using a fluorescence microscope (**P**). Total original magnification, ×100. Bound monocytes from multiple wells/group were quantified using ImageJ software (**Q**). *P < 0.05; ***P < 0.001 as determined by 1-way ANOVA and Sidak’s multiple-comparison test (versus siNC control, *n* = 25–29). (**R**) Phagocytosis assays were performed with fluorescently labeled *E*. *coli* bioparticles in THP1 macrophages transfected with siDR or siNC and treated with IL-4 (20 ng/mL) or LPS (100 ng/mL) for 24 hours. Results shown as fluorescence from phagocytosed particles. **P < 0.01; ***P < 0.001 as determined by multiple unpaired *t* tests, with correction for multiple comparisons using the Holm-Sidak method (versus siNC, *n* = 6).

We next examined the effect of *DRAIR* on monocyte gene expression and the inflammatory phenotype using both gain-of-function (overexpression) and loss-of-function (gene silencing) approaches. For overexpression experiments, we cloned *DRAIR* cDNA into a lentiviral vector. Then, we transduced THP1 monocytes with lentiviruses expressing *DRAIR* or a control *EGFP*, and the gene expression was analyzed by qPCR. Results showed that *DRAIR* overexpression (Figure 3A) upregulated the nearby *CPEB2* gene and the antiinflammatory gene *IL1RN* (Figure 3, B and C), but it downregulated proinflammatory genes *TNF* and *FCGR3B* (*CD16b*) (Figure 3, D and E). Furthermore, *DRAIR* overexpression also upregulated macrophage markers such as scavenger receptors *CD68* and *CD36*, as well as a BCL2 family member *MCL1* that regulates apoptosis (Figure 3, F–H). These results demonstrate that lncRNA *DRAIR* may regulate macrophage functions and promote antiinflammatory processes.

Next, we determined the effect of *DRAIR* silencing on the monocyte inflammatory phenotype. THP1 cells were transfected with siRNAs targeting *DRAIR* (siDR) or negative control siRNA (siNC) and, 48 hours later, treated with LPS (100 ng/mL) for 24 hours. qPCR analysis showed that siDR inhibited *DRAIR* expression and nearby *CPEB2*, as well as *IL1RN*, but enhanced proinflammatory *IL1B* expression (Figure 3, I–L). We also tested whether *DRAIR* gene silencing can downregulate *IL1RN* expression in THP1 macrophages. THP1 cells were transfected with siDR or siNC, followed by treatment with PMA (20 ng/mL), to induce macrophage differentiation. PMA treatment increased *DRAIR* expression, along with *IL1RN* and *CPEB2*, but *DRAIR* gene silencing by siDR significantly inhibited both *CPEB2* and *IL1RN* relative to siNC transfected cells (Figure 3, M–O), further verifying that *DRAIR* can positively regulate antiinflammatory genes in macrophages.

We also examined whether *DRAIR* gene silencing can enhance monocyte-endothelial cell (monocyte-EC) adhesion, a key indicator of inflammation. THP1 monocytes were transfected with siDR or siNC and, 48 hours later, treated with or without TNF-α (10 ng/mL for 3hours). Then, THP1 monocytes were fluorescently labeled with DAPI and incubated with primary human umbilical vein ECs plated in 24-well plates to perform adhesion assays. Monocyte-EC adhesion was significantly enhanced after *DRAIR* knockdown (Figure 3, P and Q; siNC versus siDR). However, TNF-α–induced increases in monocyte-EC adhesion was not further enhanced by siDR (Figure 3, P and Q; siNC + TNF versus siDR + TNF). These results demonstrate that *DRAIR* gene silencing promotes the inflammatory phenotype in THP1 monocytes.

Phagocytosis is an important function of macrophages in normal and pathological conditions. Because *DRAIR* is induced during macrophage differentiation and because phagocytosis-related pathways were enriched in differentially regulated genes inT2D monocytes (Supplemental Figure 1G), we tested if *DRAIR* knockdown affects phagocytosis. We found that *DRAIR* knockdown with siDR significantly attenuated basal- and IL-4–induced phagocytosis of fluorescently labeled *E*. *coli* bioparticles in THP1 macrophages (Figure 3R). Furthermore, LPS also inhibited phagocytosis, and this was further inhibited after *DRAIR* knockdown (Figure 3R). Together, these data clearly demonstrate that *DRAIR* regulates antiinflammatory processes and key macrophage functions.

### DRAIR knockdown amplifies and DRAIR overexpression attenuates inflammatory genes in CD14^+^ monocytes.

**Figure 4:**
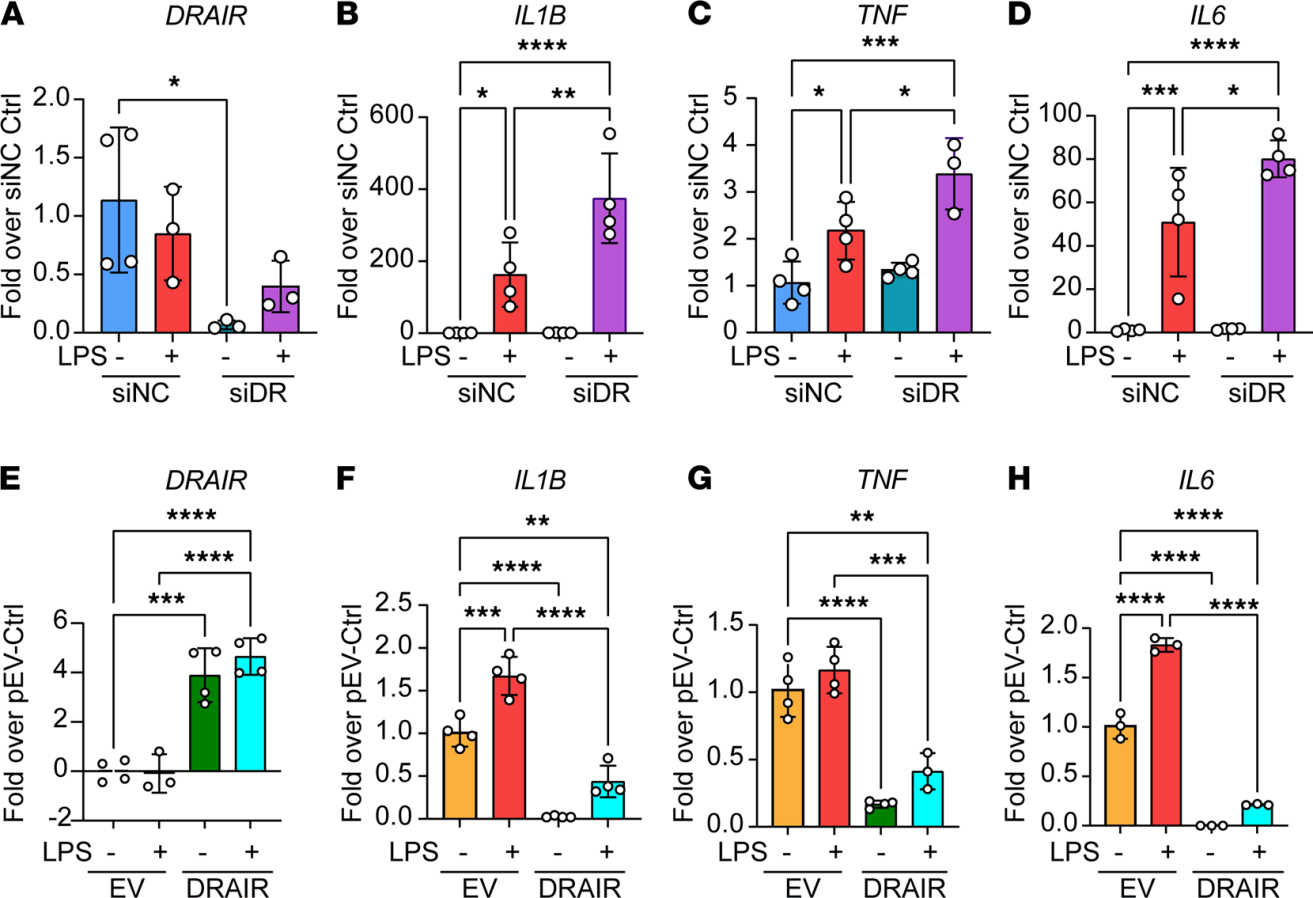
*DRAIR* regulates inflammatory phenotype of primary human CD14^+^monocytes. (**A**–**D**) *DRAIR* knockdown enhances inflammatory gene expression in human CD14^+^ monocytes. qPCR analysis of indicated genes in CD14^+^ monocytes transfected with siDR or siNC. Two days after transfection, cells were treated with or without LPS (100 ng/mL) for 24 hours and gene expression analyzed by qPCR. Results expressed as fold over siNC control (Ctrl). *P < 0.05; **P < 0.01; ***P < 0.001; ****P < 0.0001 versus siNC-LPS, as determined by 1-way ANOVA followed by Sidak’s multiple-comparison tests (*n* = 4). Similar results were obtained with CD14^+^ monocytes isolated from 2 other volunteers. (**E**–**H**) *DRAIR* overexpression inhibits inflammatory phenotype in primary human CD14^+^ monocytes. CD14^+^ monocytes transfected with plasmid pDRAIR expressing DRAIR (DRAIR) or empty vector pcDNA3.1 (EV) were treated with LPS (100 ng/mL) for 24 hours, and expression of indicated genes was analyzed by qPCR (*n* = 4). **P < 0.01; ***P < 0.001; ****P < 0.0001 as determined by 1-way ANOVA and Sidak’s multiple-comparison tests (*n* = 4).

Next, we examined if DRAIR can elicit similar antiinflammatory effects in human CD14^+^ monocytes. We differentiated CD14^+^ monocytes isolated from nondiabetic human volunteers and transfected them with siDR or siNC. Two days after transfection, cells were treated with or without LPS, and gene expression was analyzed. siDR-mediated knockdown of *DRAIR* significantly enhanced LPS-induced expression of *IL1B*, *TNF*, and *IL6* (Figure 4, A–D). Conversely, we examined the consequences of *DRAIR* overexpression by transfecting CD14^+^ monocytes with a plasmid vector pDRAIR expressing *DRAIR* or control empty vector pcDNA3.1. DRAIR overexpression significantly attenuated LPS-induced *IL1B,* *TNF*, and *IL6* expression (Figure 4, E–H), clearly demonstrating that *DRAIR* mediates antiinflammatory effects even in primary human monocytes.

### DRAIR expression is induced by macrophage differentiation and by antiinflammatory cytokines via KLF4.

**Figure 5:**
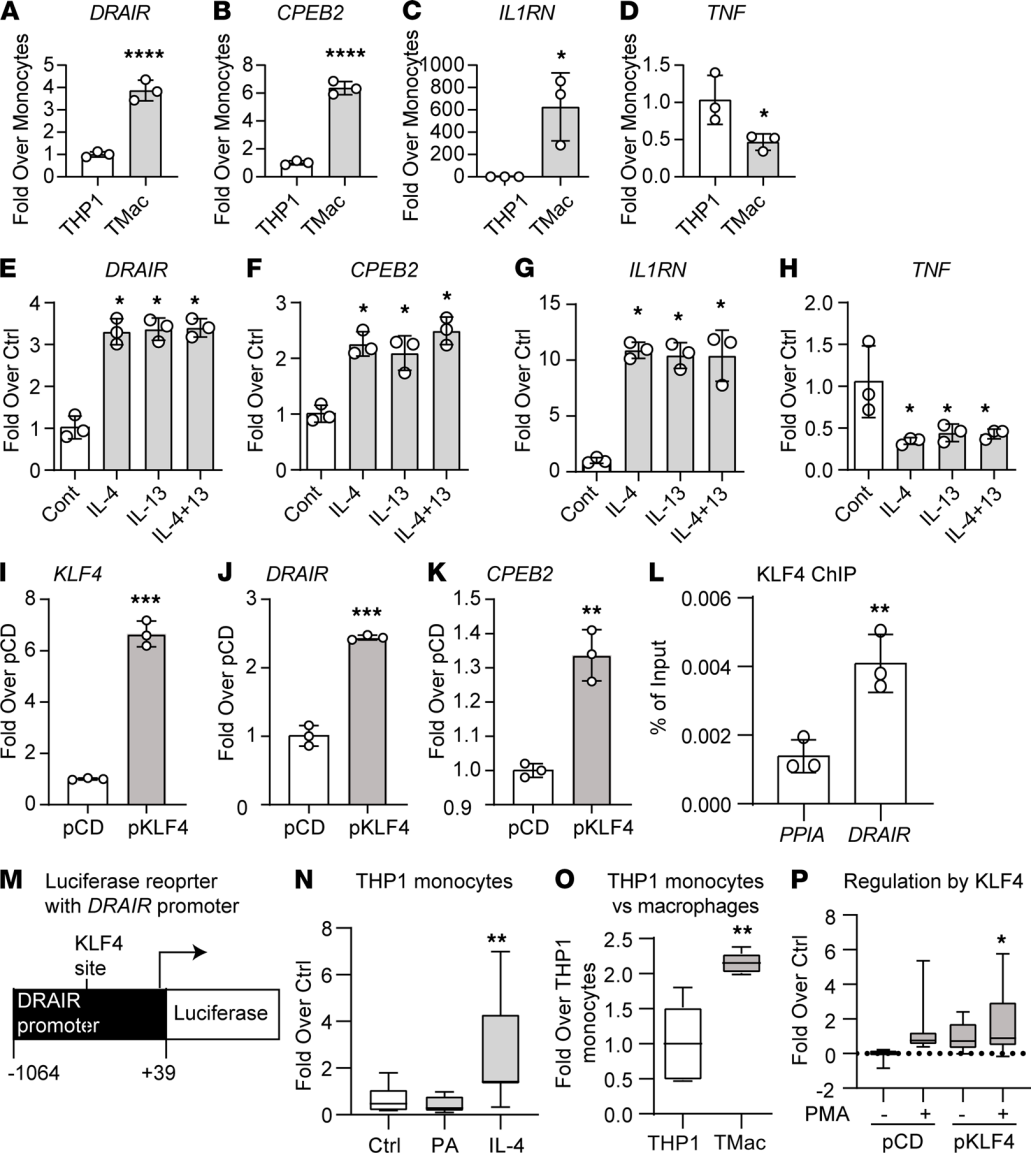
*DRAIR* is regulated during macrophage differentiation and by antiinflammatory cytokines. (**A**–**D**) qPCR analysis of indicated genes in THP1 monocytes (THP1) before and after differentiation into macrophages (TMac) with PMA (20 ng/mL) for 24 hours. *P < 0.05; ****P < 0.0001 as determined by unpaired *t* test (*n* = 3). (**E**–**H**) Expression of indicated genes in THP1 macrophages treated with IL-4 or IL-13 or a combination of both (20 ng/mL each). *P < 0.05 (*n* = 3) as determined by 1-way ANOVA and Dunnett’s multiple comparisons tests. (**I**–**K**) Gene expression analysis in THP1 cells transiently transfected with control pcDNA3.1 (pCD) or KLF4 expression (pKLF4) plasmids. Gene expression analyses were performed 48 hours after transfection. **P < 0.01; ***P < 0.001 versus pCD as determined by unpaired *t* test (*n* = 3). (**L**) ChIP-qPCR analysis of ChIP assays with KLF4 antibody with indicated promoter primers (**P < 0.01 versus *PPIA* promoter, *n* = 3). (**M**) Schematic of the reporter plasmid (pDRluc) with *DRAIR* promoter cloned upstream of firefly luciferase reporter gene. KLF4 site (–760) in *DRAIR* promoter (not to scale). (**N**–**P**) Luciferase activities with THP1 cells cotransfected with pDRluc and internal control Renilla luciferase. In addition, plasmids pKLF4 and pCDNA3.1 were also cotransfected in **P**. One day after transfection, cells were treated as indicated for 24 hours. Luciferase activities are reported as fold over controls. In **N**, Ctrl, control; PA, palmitic acid (200 μM); IL-4, 20 ng/mL; in **O**, PMA-PMA 20 ng/mL. *P < 0.05, **P < 0.01 as determined by 1-way ANOVA followed by Dunnett’s multiple-comparison test (**N** and **P**, *n* = 10–13) and unpaired *t* test for **O** (*n* = 5).

Monocyte to macrophage differentiation plays an important role in inflammation and homeostasis. Therefore, we examined the expression of *DRAIR* in THP1 cells that were differentiated into macrophages by treatment with PMA (20 ng/mL). We found that expressions of *DRAIR* and *CPEB2* were significantly increased in THP1 macrophages relative to monocytes at 24 hours after PMA treatment (Figure 5, A and B). In parallel, antiinflammatory *IL1RN* was upregulated and proinflammatory *TNF* was downregulated (Figure 5, C and D). Next, *DRAIR* expression was examined after treatment with IL-4 and IL-13, which are known to promote alternatively activated (M2) phenotype and antiinflammatory responses in macrophages. Both IL-4 and IL-13 (20 ng/mL, 24 hours) significantly induced *DRAIR* expression (Figure 5E). In parallel, *CPEB2* and *IL1RN* were also upregulated by these 2 cytokines, whereas *TNF* was downregulated (Figure 5, F–H). Treatment with a mixture of IL-4 and IL-13 had no further additive effects, suggesting that similar pathways regulate these genes (Figure 5, E–H).

A TF motif search of the *DRAIR* promoter by TRANSFAC analysis revealed several binding sites for Kruppel-like family (KLF) members, including KLF4 (Supplemental Figure 5), a reported negative regulator of the macrophage inflammatory phenotype (31). Accordingly, transfection of THP1 cells with a KLF4 expression plasmid (pKLF4) significantly increased *KLF4* expression and upregulated *DRAIR* and *CPEB2* relative to empty vector (pCD) (Figure 5, I–K). Furthermore, ChIP assays with KLF4 antibody confirmed KLF4 binding at the *DRAIR* promoter but not at control *PPIA* promoter (Figure 5L). To further verify whether KLF4 regulates the *DRAIR* promoter, we constructed a reporter plasmid pDRluc in which LUC is expressed from the *DRAIR* promoter region (–1064 to +39 bp) harboring the KLF4 binding site at –760 bp (Figure 5M). Transient transfection with pDRluc showed that *DRAIR* promoter activity was inhibited by PA (200 μM) but transactivated by IL-4 (20 ng/mL) and PMA that promotes differentiation of THP1 monocytes (THP) into macrophages (TMac) (Figure 5, N and O). Furthermore, cotransfection of pDRluc reporter plasmid with pKLF4 plasmid induced transactivation of the *DRAIR* promoter relative to pCD, which was further enhanced after PMA treatment (Figure 5P). These results demonstrate that *DRAIR* expression is upregulated during macrophage differentiation and by antiinflammatory cytokines and that KLF4 plays a key role in its transcriptional activation.

### CPEB2 gene silencing also promotes the inflammatory phenotype in THP1 monocytes.

**Figure 6:**
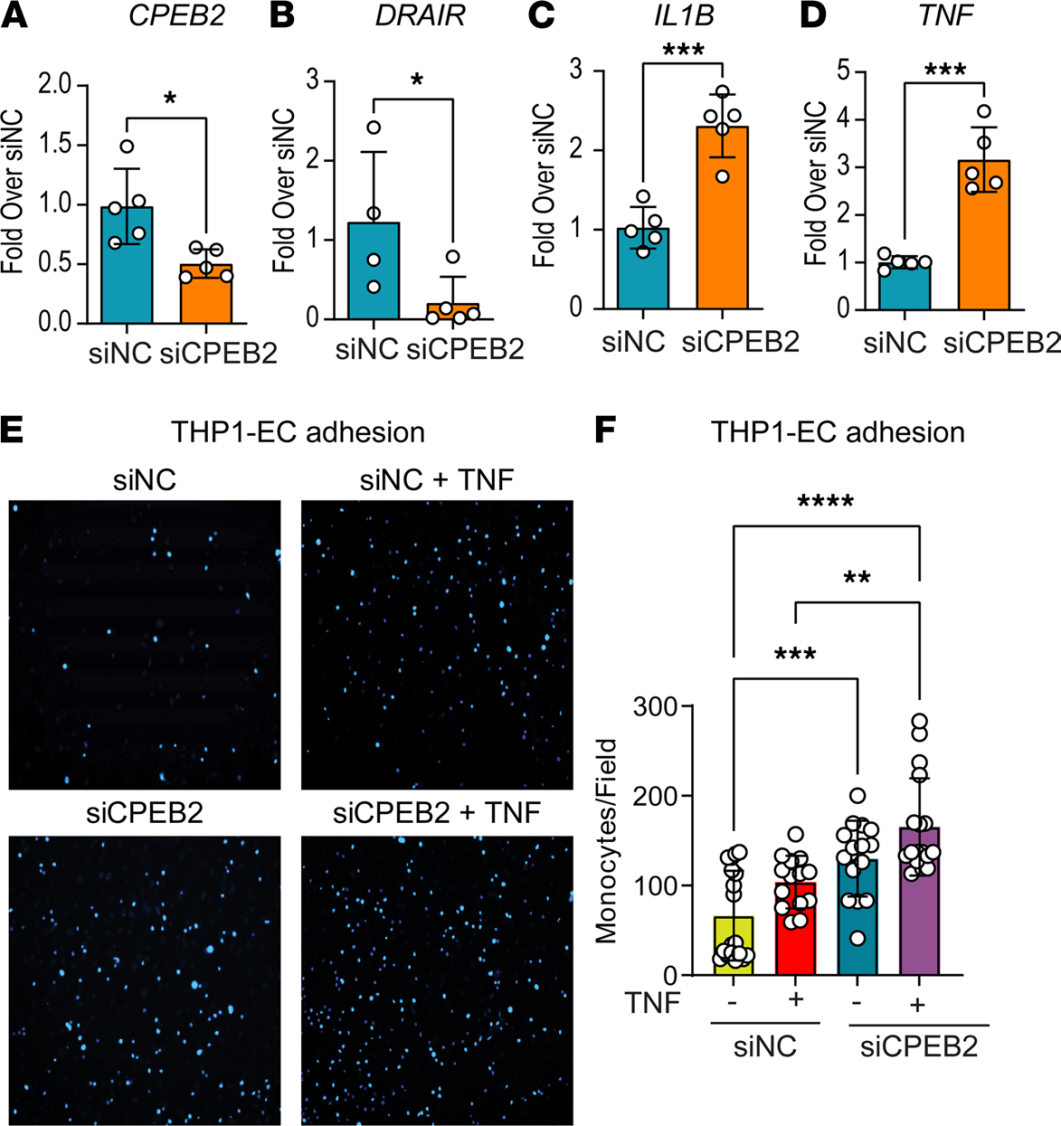
*CPEB2* knockdown also promotes inflammatory phenotype in THP1 monocytes. (**A**–**D**) Expression of indicated genes was analyzed by qPCR in THP1 cells transfected with siNC and siRNA targeting *CPEB2* (siCPEB2). *P < 0.05; ***P < 0.001 (*n* = 5) versus untreated siNC as determined by unpaired *t* test. (**E** and **F**) Images and quantification of monocyte–endothelial cell (monocyte-EC) adhesion assays. THP1 monocytes transfected with siCPEB2 or siNC were treated ± TNF-α (10 ng/mL, 3 hours), fluorescently labeled with DAPI, and incubated with EC plated in 24-well plates. EC monolayers were washed with PBS, and images were collected using a fluorescence microscope. Total original magnification, ×100. Bound monocytes (blue color spots) were counted using ImageJ software. **P < 0.01, ***P < 0.001, ****P < 0.0001 versus untreated siNC and versus untreated siCPEB2 as determined by 1-way ANOVA and Sidak’s multiple-comparison test (*n* = 16–19).

Because our data suggest that *DRAIR* could regulate the nearby gene *CPEB2* that codes for CPEB2 protein, we examined if *CPEB2* knockdown mimics the antiinflammatory effects of DRAIR. Transfection of THP1 cells with siRNA targeting *CPEB2* (siCPEB2) inhibited expression of both *CPEB2* and *DRAIR*, but upregulated *IL1B* and *TNF* compared with siNC transfected cells (Figure 6, A–D). Furthermore, *CPEB2* knockdown in THP1 cells also enhanced both basal- and TNF-α–induced monocyte-EC adhesion (Figure 6, E and F). These results suggest that CPEB2 may mediate some of the antiinflammatory effects of *DRAIR* in THP1 cells.

### ChIRP analysis reveals DRAIR binding sites on chromatin.

**Figure 7:**
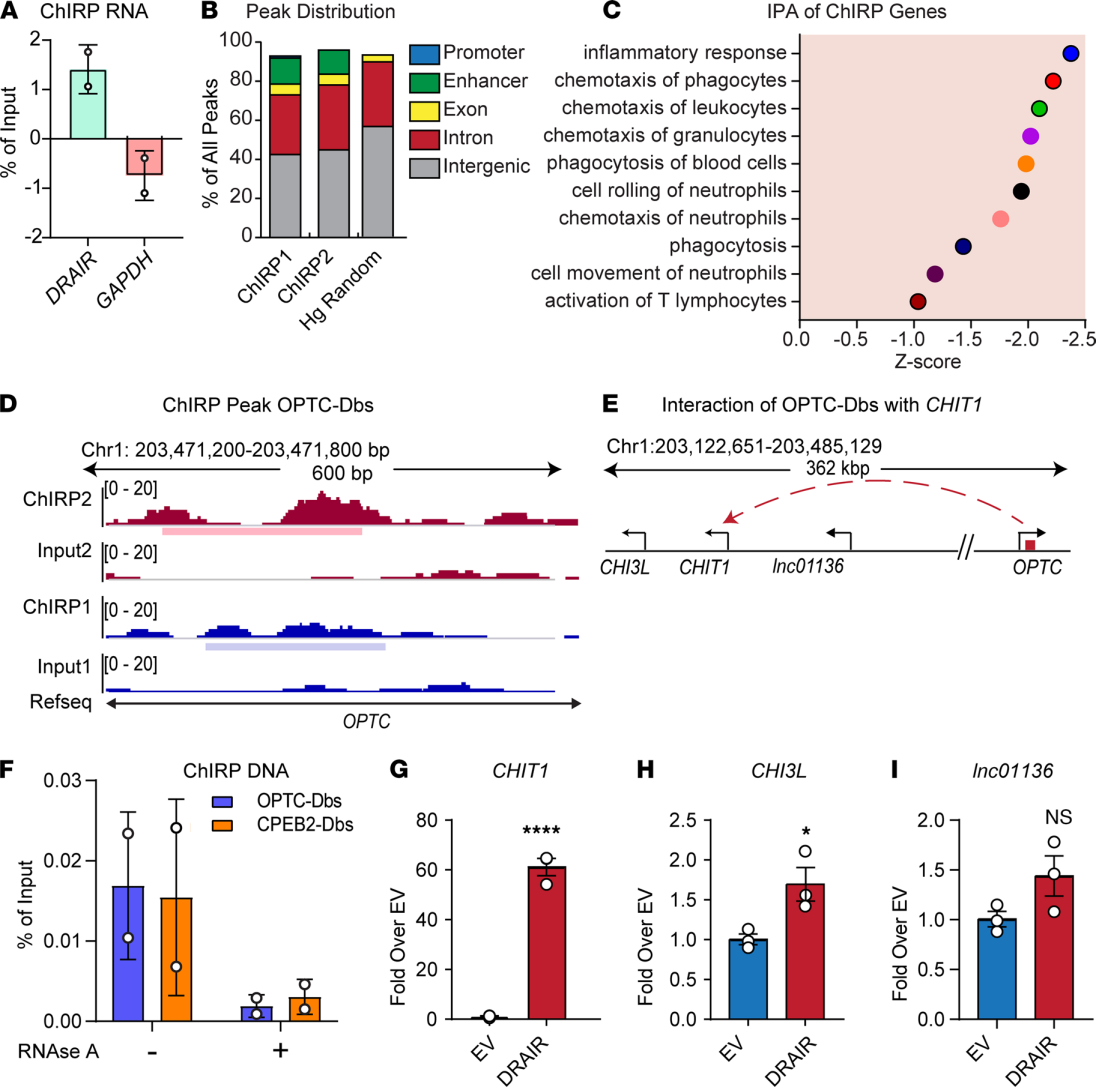
ChIRP analysis reveals *DRAIR* binding sites on chromatin. (**A**) qPCR analysis of RNA from ChIRP assays with indicated primers. Percentage of input values from 2 experiments were log transformed. ChIRP assays were performed using biotinylated *DRAIR* antisense oligonucleotides in THP1 cells. RNA-recovered from ChIRP assays was analyzed by qPCR and DNA by DNA sequencing (ChIRP-seq). (**B**) Genomic distribution of *DRAIR* binding sites (Dbs) identified from ChIRP-seq analysis. (**C**) GO terms enriched in genes nearby (±250 kb) Dbs as determined by IPA. (**D** and **E**). Schematic of Dbs in the intronic region of *OPTC* gene (OPTC-Dbs) (**D**) and its potential interaction with upstream region of *CHIT1* gene (**E**) identified using Hi-C plotter (CHiCP) tool. ChIRP1 and ChIRP2 in **B** and **D** refer to duplicates. (**F**) ChIRP-qPCR analysis of ChIRP-DNA using primers for indicated DRAIR binding sites. THP1 cell lysates were treated ± pancreatic ribonuclease A (RNase A), and ChIRP assays were performed with biotinylated *DRAIR* probes. RNase A–treated samples served as negative controls. Percentage of input values from 2 experiments were log transformed. (**G**–**I**) qPCR analysis of indicated genes in THP1 cells overexpressing *DRAIR* versus control vector (EV). *P < 0.05; ****P < 0.0001 versus EV as determined by unpaired *t* test (*n* = 3).

Interactions with chromatin and chromatin-interacting proteins are major mechanisms by which nuclear lncRNAs can regulate gene expression. Because *DRAIR* is enriched in chromatin, we examined the interactions of *DRAIR* with chromatin by performing chromatin isolation by RNA purification (ChIRP) assays with THP1 nuclear lysates using biotinylated tiling oligonucleotide probes targeting the *DRAIR* RNA. Quantitative PCR (qPCR) confirmed specific recovery of *DRAIR* transcript but not control *GAPDH* in RNA recovered from ChIRP assays (Figure 7A). DNA recovered from ChIRP assays (ChIRP-DNA) was analyzed by DNA-seq (ChIRP-seq) to identify genome-wide interactions of *DRAIR*. ChIRP-seq analysis revealed 152 *DRAIR* binding sites (Dbs) on multiple chromosomes in THP1 cells (Supplemental Table 2). These Dbs were enriched at promoter, intronic, and enhancer regions on the chromatin (Figure 7B). Interestingly, 290 genes located nearby Dbs (± 250 kb; Supplemental Table 3) were differentially expressed in our RNA-seq data from T2D monocytes (Figure 1B). IPA showed that these genes nearby Dbs are involved in inflammatory response, chemotaxis, and phagocytosis (Figure 7C). Transfac analysis of Dbs showed enrichment of key TFs involved in monocyte and macrophage functions, including members of the KLF family (Supplemental Figure 6).

To further understand the functions of *DRAIR* at the chromatin level, we examined the potential interaction of Dbs with other genomic regions using capture Hi-C plotter (CHiCP), a publicly available promoter capture Hi-C database in human cells (32). CHiCP analysis of human macrophages revealed interactions of Dbs with multiple genomic regions, suggesting their role in *DRAIR* mediated gene regulation. One of the Dbs was located in the intronic region of *OPTC* gene on chromosome 1, which we named OPTC-Dbs (Figure 7D). CHiCP analysis revealed that the OPTC-Dbs potentially interacts with a genomic region 362 kb away on the same chromosome with a Capture Hi-C Analysis of Genomic Organization (CHiCAGO) score of > 5. This Dbs interacting region is upstream of *CHIT1* and *CHI3L* genes (Figure 7E), which regulate the macrophage M2 phenotype (33).

Using candidate ChIRP-qPCR, we validated the interaction between *DRAIR* and the OPTC-Dbs in THP1 monocytes. Moreover, this interaction was abolished by RNase treatment of THP1 lysates, confirming the specificity of the interaction (Figure 7F). We next examined whether *DRAIR* regulates *CHIT1* and *CHI3L* genes. qPCR analysis showed that *DRAIR* overexpression significantly upregulated *CHIT1* and *CHI3L,* but not *lnc01136*, a lncRNA expressed in the nearby region (Figure 7, G–I), suggesting that *DRAIR* may regulate these genes through interaction at OPTC-Dbs in monocytes. In addition, candidate ChIRP-qPCR also demonstrated *DRAIR* interaction with the promoter of adjacent *CPEB2* (Figure 7F), suggesting a role for chromatin interaction in the regulation of nearby *CPEB2* gene by *DRAIR*. These results indicate that *DRAIR* interactions with chromatin may play important roles in the regulation of proximally and distally located monocyte/macrophage genes.

### DRAIR interacts with G9a histone methyltransferase and controls repressive histone modifications at target gene promoters.

**Figure 8:**
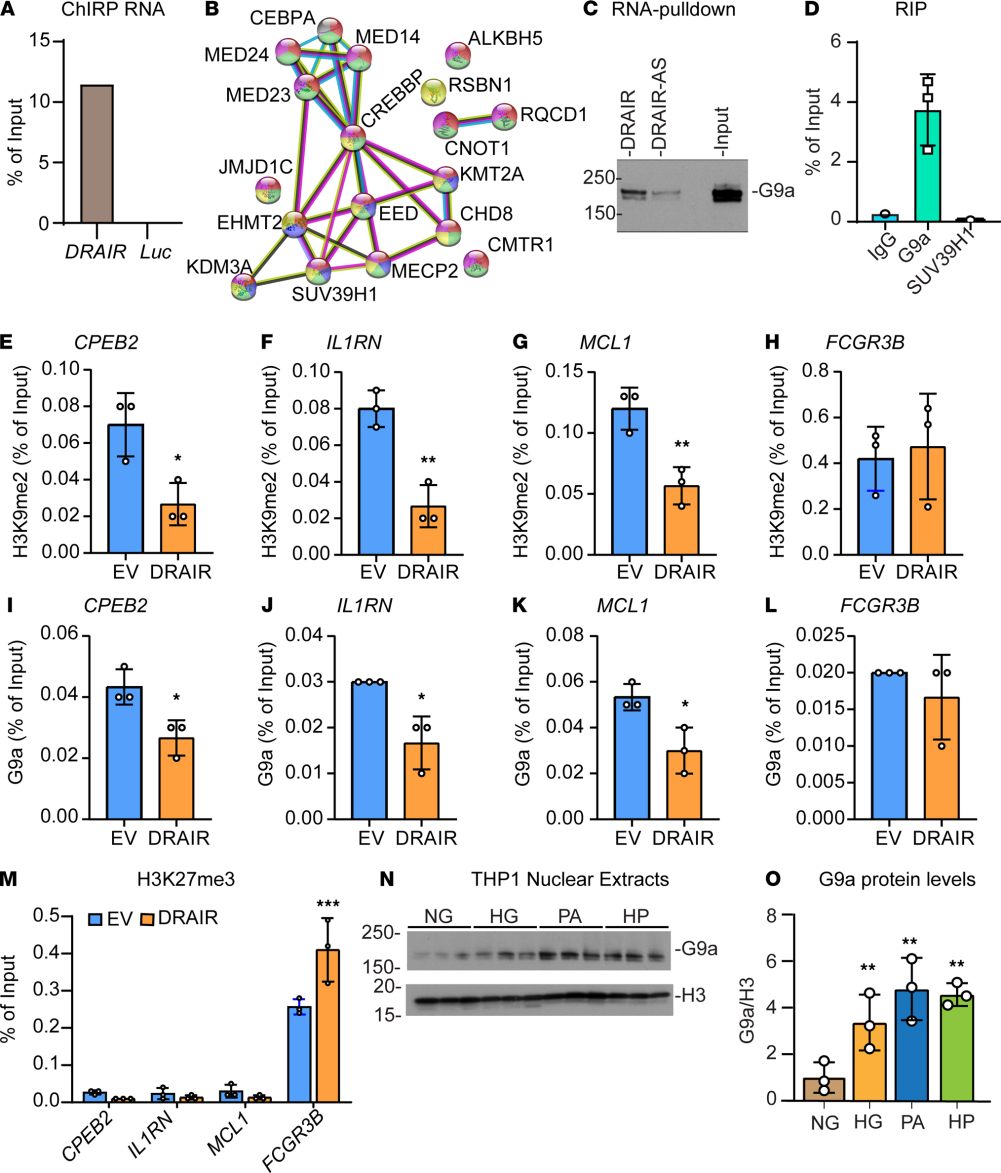
*DRAIR* interacts with G9a and alters levels of repressive histone modifications. (**A**) qPCR of RNA recovered from ChIRP with *DRAIR* probes or negative control luciferase (LUC) probes. Cell lysates from THP1 monocytes overexpressing *DRAIR* were subjected to ChIRP assays with biotinylated *DRAIR* probes and negative control biotinylated LUC probes. ChIRP complexes were captured on streptavidin beads, and RNA from an aliquot of beads was analyzed by qPCR with *DRAIR* primers. (**B**) The nucleic acid–protein complexes from ChIRP were fractionated on SDS-PAGE and subjected to mass spectrometry to identify proteins interacting with *DRAIR* probes. STRING analysis of indicated *DRAIR*-interacting proteins identified by ChIRP mass spectrometry (Supplemental Figure 7). Colors represent GO biological functions shown in Supplemental Figure 7. (**C**) Immunoblotting of proteins from RNA pulldown assays using *DRAIR* and *DRAIR* antisense (DRAIR-AS) probes with G9a antibody. (**D**) qPCR analysis of RNA recovered after RNA IP with indicated antibodies. (**E**–**M**) ChIP-qPCR analysis of DNA recovered from ChIP assays using lysates from THP1 cells overexpressing *DRAIR* and empty vector pcDNA3.1 (EV) with antibodies against H3K9me2 (**E**–**H**), G9a (**I**–**L**), and H3K27me3 (**M**) using indicated gene promoter primers. (**N** and **O**) Immunoblotting of THP1 nuclear extracts with indicated antibodies (**N**) and quantification of G9a in nuclear extracts (**O**). THP1 cells were treated with NG (5.5 mM glucose) and HG (25 mM glucose) for 72 hours. Palmitic acid (PA, 200 μM) was also added in the final 24 hours to NG- (PA) and HG-treated (HP) cells. *P < 0.05; **P < 0.01; ***P < 0.001 (*n* = 3) as determined by unpaired *t* test (**E**–**L**) and 2-way ANOVA with Sidak’s (**M**), and 1-way ANOVA with Dunnett’s (**O**) multiple-comparison testing.

We next used ChIRP followed by mass spectrometry (ChIRP-MS) to explore whether *DRAIR* also regulates gene expression via interactions with chromatin modifying enzymes/proteins. Because endogenous *DRAIR* is not abundant in THP1 monocytes cells, we used a THP1 cell line stably overexpressing *DRAIR* for these experiments. ChIRP was performed using biotinylated tiling oligonucleotide probes complementary to DRAIR, and *LUC* transcript (negative control). qPCR of RNA recovered from ChIRP samples (ChIRP-RNA) showed enrichment of *DRAIR* RNA only with DRAIR probes but not with LUC probes, confirming specificity (Figure 8A). Following ChIRP, nucleic acid–protein complexes were fractionated by SDS-PAGE (4%–15%), and the proteins captured using DRAIR or Luc probes were analyzed by MS. ChIRP-MS analysis revealed that *DRAIR* interacts with several nuclear proteins including histone modifying enzymes, transcription repressors, and enhancer interacting proteins (Supplemental Figure 7A). STRING analysis (34) of *DRAIR*-interacting proteins showed that networks of these proteins were associated with GO biological processes and molecular functions related to histone methylation, transcription, chromatin organization, and gene expression (Figure 8B and Supplemental Figure 7, B and C). These results suggest that *DRAIR* interaction with chromatin modifying protein networks may regulate epigenetic mechanisms involved in *DRAIR*-mediated gene regulation.

To examine this further, we validated *DRAIR* interaction with 2 proteins identified by ChIRP-MS, namely histone methyl transferases G9a (EHMT2) and SUV39H1 (KMT1A), which mediate the repressive histone modifications histone H3 lysine-9 dimethylation (H3K9me2) and H3K9me3, respectively. Previous studies showed dysregulation of H3K9me2 and H3K9me3 by hyperglycemia and diabetes in monocytes and vascular cells (11, 35). *DRAIR* interaction with G9a was validated using RNA pulldown and RNA IP (RIP) assays. RNA pulldown was performed on nuclear extracts from THP1 cells using a biotinylated *DRAIR* sense probe and *DRAIR* antisense (DRAIR-AS) probe as negative control. Western blot analysis of RNA pulldown proteins revealed that G9a strongly interacts with DRAIR probe but not with DRAIR-AS probe (Figure 8C). However, SUV39H1 protein was not detected after RNA pulldown (data not shown). Furthermore, RIP also confirmed enrichment of *DRAIR* with G9a antibody but not with IgG or SUV39H1 antibody (Figure 8D).

Next, we determined the effect of *DRAIR* overexpression on G9a occupancy and enrichment levels of the corresponding repressive histone modification H3K9me2 at candidate *DRAIR* target genes shown in Figure 3, B–H. ChIP-qPCRs showed that H3K9me2 levels were significantly reduced at *IL1RN*, *CPEB2*, and *MCL1* promoters (Figure 8, E–G) in *DRAIR*-overexpressing cells versus empty vector pcDNA3.1 transfected cells. These candidate genes were upregulated in *DRAIR*-overexpressing cells (Figure 3). However, H3K9me2 levels were not altered at the *FCGR3B* promoter (Figure 8H). Furthermore, ChIP assays with G9a antibody showed that, in parallel, G9a occupancy was also significantly reduced at *Il1RN*, *CPEB2*, and *MCL1* promoters but not at the *FCGR3B* promoter (Figure 8, I–L). Because H3K9me2 and G9a were not altered at the *FCGR3B* promoter and because ChIRP-MS showed interaction of *DRAIR* with EED protein, which is a part of the polycomb repressive complex-2 (PRC2) that regulates H3K27me3 repressive mark (36), we tested the impact of *DRAIR* overexpression on H3K27me3. Indeed, ChIP assays showed that *DRAIR* overexpression significantly increased H3K27me3 at the *FCGR3B* promoter (Figure 8M), whose expression was decreased under these conditions (Figure 3E). These results suggest that *DRAIR* upregulates key target genes in part via sequestration of G9a, and downregulates other genes in part via activation of the PRC2 repressive complex in monocytes, thus implicating epigenetic mechanisms of action.

Interestingly, our RNA-seq data reveal that G9a gene (*EHMT2*) expression is upregulated in T2D monocytes versus controls (log_2_-fold = 1.15, FDR = 0.047, *n* = 5 each). Therefore, we examined if G9a protein levels were altered in THP1 monocytes under diabetic conditions. Immunoblot analysis of nuclear extracts from THP1 monocytes treated with HG or PA alone or with HP showed significant increase in G9a protein levels (Figure 8, N and O). Furthermore, *EHMT2* gene silencing with siRNA (siG9a) significantly increased expression of DRAIR target genes *IL1RN*, *CPEB2*, and *MCL1* relative to nontargeting siRNA (siNTC) in THP1 cells (Supplemental Figure 8). These data suggest that downregulation of *DRAIR* and upregulation of G9a in T2D might work together, at least in part, to repress key antiinflammatory genes.

### Mouse orthologous Drair is downregulated in macrophages from T2D mice and regulates the inflammatory phenotype.

**Figure 9:**
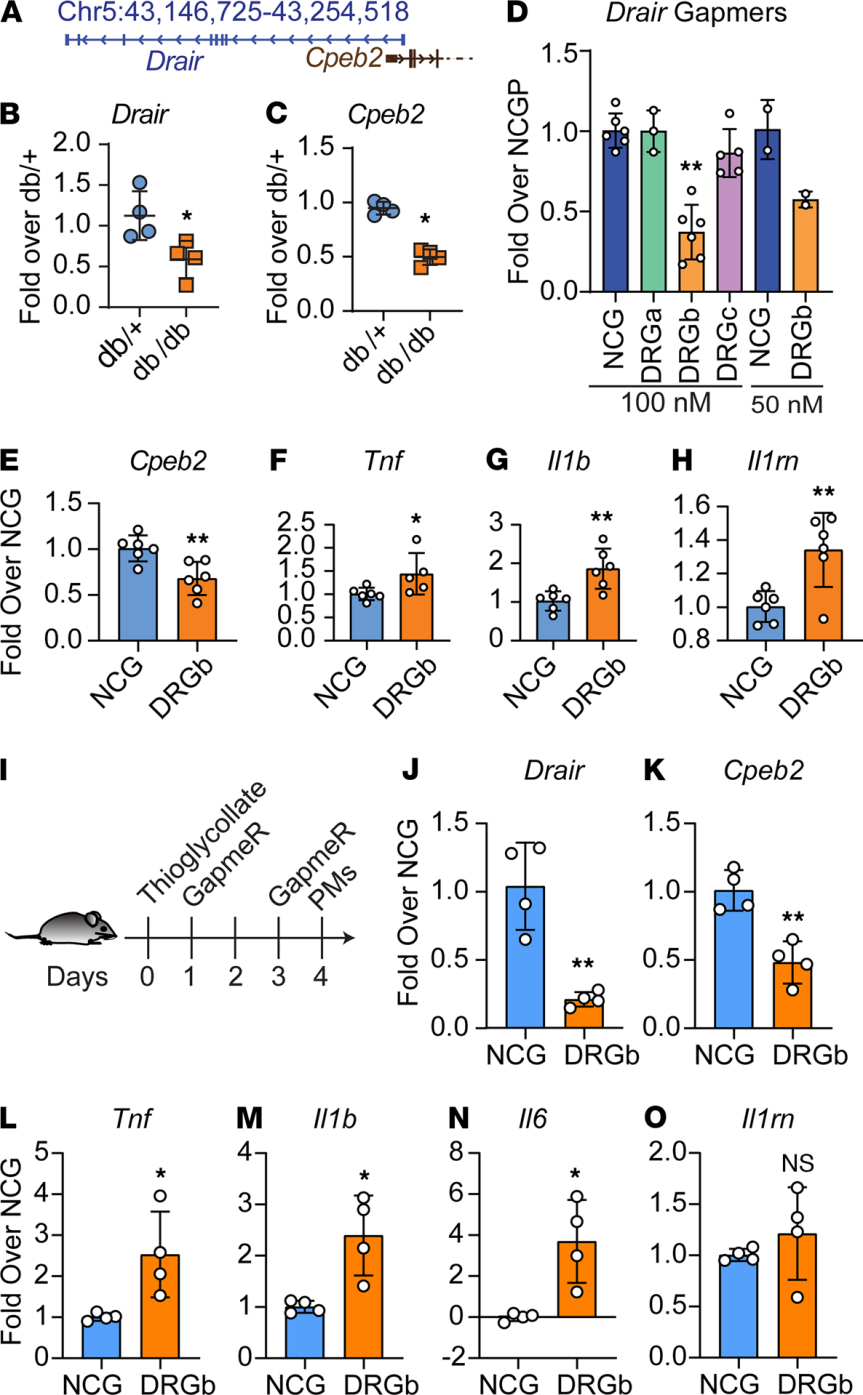
Mouse orthologous *Drair* is downregulated in macrophages from T2D mice and regulates inflammatory phenotype in macrophages. (**A**) Genomic organization of *Drair* and *Cpeb2* in the mouse genome (mm9). (**B** and **C**) qPCR analysis of *Drair* in peritoneal macrophages (PMs) from type2 diabetic *db/db* mice versus genetic control *db/+* mice. *P < 0.05 as determined by unpaired *t* test (*n* = 4). (**D**) qPCR analysis of RAW cells transfected with control (NCG) and indicated *Drair* GapmeRs (DRGa, DRGb, and DRGc) (*n* = 2–6). (**E**–**H**) qPCR analysis of indicated genes after *Drair* knockdown with DRGb GapmeR (DRGb) versus NCG GapmeR in RAW cells *P < 0.05; **P < 0.01 (paired *t* test). (**I**) Experimental design for *Drair* knockdown in C57BL/6 mice with GapmeRs (NCG or DRGb). (**J**–**O**) Gene expression analysis in PMs from C57BL/6 mice treated with NCG or *Drair* (DRGb) GapmeRs. *P < 0.05; **P < 0.01 versus NCG as determined by unpaired *t* test (*n* = 4).

To evaluate putative conservation across species, we next examined expression and function of mouse orthologous *Drair*. Using liftOver, we found that an annotated lncRNA *Gm7854* on mouse chromosome 5 is expressed as a divergent transcript near the mouse *Cpeb2* gene (Figure 9A) similar to the human transcript. We designated *Gm7854* as mouse *Drair* and examined its expression in peritoneal macrophages (PMs) from *db/db* mice, a well-known mouse model of T2D. qPCR showed that *Drair* and nearby *Cpeb2* were significantly downregulated in PMs from *db/db* mice versus nondiabetic control *db/+* mice (Figure 9, B and C), similar to human *DRAIR*. We next examined the outcome of targeting mouse *Drair* with specific locked nucleic acid–modified (LNA-modified) GapmeRs. Transfection of the mouse RAW264.7 macrophage cell line with 3 GapmeRs (DRGa, DRGb, and DRGc) targeting different regions of *Drair* showed that only DRGb significantly inhibited *Drair* expression relative to control GapmeR NCG (Figure 9D). Moreover, *Drair* knockdown inhibited *Cpeb2* but upregulated *Tnf* and *Il1b* in RAW264.7 macrophages (Figure 9, E–G) compared with NCG. Interestingly, the expression of *Il1rn* was also increased (Figure 9H), possibly as a feedback response to increased inflammation.

Next, we examined the effect of *Drair* knockdown in vivo on the macrophage inflammatory phenotype. Because, *Drair* is already downregulated in diabetic *db/db* mouse macrophages, we examined whether *Drair* knockdown in nondiabetic C57BL/6 mice can elicit a diabetic-like inflammatory phenotype in macrophages. C57BL/6 mice were injected i.p. with thioglycollate (3%) to induce inflammation and subsequently injected i.p. with 2 doses (5 mg/kg) of in vivo grade *Drair* GapmeR DRGb or negative control GapmeR NCG (Figure 9I). Gene expression analyzed in PMs 24 hours after the second dose showed that GapmeR DRGb significantly reduced *Drair* expression relative to GapmeR NCG–injected mice (Figure 9J). Furthermore, *Drair* knockdown inhibited the expression of nearby *Cpeb2* (Figure 9K)and, in parallel, increased the expression of proinflammatory genes *Tnf*, *Il1b*, and *Il6* (Figure 9, L–N), whereas *Il1rn* expression was not altered (Figure 9O). Altogether, these data demonstrate that *Drair* has similar antiinflammatory functions in mice and human macrophages

## Discussion

Here, we show that several antiinflammatory genes and antiproliferative genes are downregulated in monocytes from T2D individuals relative to healthy controls and that this downregulation may contribute to increased inflammatory phenotype and monocyte numbers. Notably, we demonstrate that levels of a key lncRNA, *DRAIR,* were significantly lower in monocytes from T2D subjects, as well as in primary human monocytes from nondiabetic volunteers treated with HP. Our data show that *DRAIR* can promote antiinflammatory phenotype in monocytes/macrophages and that *DRAIR* expression is regulated by the TF KLF4, which was previously identified as a negative regulator of macrophage inflammation (31). Notably, we found that *DRAIR* increases key target antiinflammatory genes, such as *IL1RN* and *CPEB2*, in monocytes through potentially novel epigenetic derepression mechanisms. In addition, we found that the mouse ortholog *Drair* is downregulated in macrophages of T2D mice and that its knock down in vivo in nondiabetic mice increases expression of inflammatory genes in macrophages. These data suggest that downregulation of lncRNAs, such as *DRAIR*, that control endogenous antiinflammatory networks may contribute to key mechanisms leading to chronic inflammatory phenotype of monocytes in T2D and its complications.

Interestingly, IPA of downregulated genes in T2D (identified from RNA-seq) showed enrichment of NF-κB and ROS pathways. Because many of the downregulated genes, including *IL1RN*, *NFKBIA*, *TNFAIP3* (A20), and *NFE2L2* (NRF2) are well-known mediators of antiinflammatory and antioxidative stress mechanisms (Figure 1C), their downregulation can augment inflammatory phenotypes. In addition, downregulation of tumor suppressors such as *PTEN* can lead to monocytosis, thus further increasing the number of inflammatory monocytes. Evidence shows that monocytosis and enhanced infiltration of inflammatory monocytes in diabetes is linked with increased atherosclerosis burden (6). Together, these data show that downregulation of key antiinflammatory and tumor suppressor/antiproliferative genes in T2D might lead to increased inflammatory and proliferative state of monocytes implicated in diabetes vascular complications.

The key antiinflammatory gene, *IL1RN*, was one of the most highly downregulated genes in T2D monocytes. Its protein product IL-1Ra is a member of the IL-1 family that binds to IL-1 receptors and inhibits their responses. IL-1Ra expression is increased by proinflammatory agents and in chronic inflammatory diseases and plays an important role in host defense against inflammation (37). Experimental evidence from animal models and clinical trials using IL-1Ra (Anakinra) have underscored its role in curbing inflammation in diabetes and atherosclerosis (38, 39). Disruption in the balance between pathological and protective factors could promote chronic inflammation (5). Our data showing *IL1RN* is downregulated in T2D monocytes support the presence of such imbalances and highlight the importance of further understanding the mechanisms and factors that inhibit or repress endogenous antiinflammatory networks in T2D.

Several lncRNAs are reported to regulate the inflammatory phenotype in macrophages (16), but the role of monocyte lncRNAs in human T2D is poorly understood. In this study, we demonstrated that hundreds of lncRNAs were differentially expressed in CD14^+^ monocytes from subjects with T2D versus controls, including multiple divergent transcripts whose functions in the monocyte/macrophage phenotype are not known. One of our key findings is that a divergent lncRNA *DRAIR* is downregulated in T2D and regulates antiinflammatory functions and genes, such as *IL1RN* and *CPEB2*, in monocytes and macrophages. *IL1RN* can be induced by several agents, including cytokines and viral and bacterial components, and it is a known antiinflammatory factor (37). *CPEB* members are regulated by inflammation and hypoxia (30, 40), but mechanisms of their regulation and action in monocytes are unclear. Our studies uncovered potentially novel lncRNA (*DRAIR*) dependent mechanisms in the regulation of *IL1RN* and *CPEB2*. In addition, we also demonstrated a potentially unknown antiinflammatory function of *CPEB2* in monocytes. Cytoplasmic localization of CPEB2 protein suggests it may act via posttranscriptional mechanisms to regulate inflammatory genes (30, 40). Interestingly, we found *CPEB2* knockdown also reduced *DRAIR* expression, suggesting that posttranscriptional regulation of *DRAIR* by CPEB2 protein may also contribute to its antiinflammatory effects.

Furthermore, we found that *DRAIR* regulates key macrophage genes (*CD68* and *CD36*) and an antiapoptotic gene *MCL1* (41) involved in macrophage differentiation, apoptosis, and phagocytosis. Because these processes play key roles in the pathophysiological functions of macrophages associated with cardiovascular disorders like atherosclerosis (3, 6), *DRAIR* downregulation in diabetes may also accelerate inflammatory cardiovascular complications. The antiinflammatory functions of *DRAIR* are further supported by our mouse in vivo data showing that *Drair* expression is downregulated in macrophages of T2D mice and that its knockdown in nondiabetic mice enhances the macrophage inflammatory phenotype. Our experimental evidence showing *DRAIR* inhibition by diabetic milieu and *DRAIR* upregulation by mediators of alternative macrophage activation, such as antiinflammatory cytokines and TF KLF4, further support the involvement of *DRAIR* in antiinflammatory processes in monocyte/macrophages.

We found that *DRAIR* is enriched in nuclear and chromatin fractions. Such nuclear lncRNAs can promote chromatin remodeling via interactions with chromatin and chromatin modifying enzymes to regulate target gene expression (16, 18). Our ChIRP-seq data demonstrate that *DRAIR* interacts with chromatin at the promoter of nearby *CPEB2*, as well as other genomic loci on multiple chromosomes. Notably, our results uncover a key DRAIR-chromatin binding site located in the *OPTC* gene (OPTC-Dbs) that interacts with the upstream region of *CHIT1* and *CHI3L* genes and might play a key role in DRAIR-induced expression of *CHIT1* and *CHI3L,* genes associated with monocyte to macrophage differentiation (33). On the other hand, our ChIRP-seq analysis did not reveal *DRAIR*-chromatin interactions upstream of other *DRAIR* target genes validated in this study, such as *IL1RN* and *MCL1*. However, several genes located near Dbs (±250 kb), including *CHIT1* discussed above, were dysregulated in T2D monocytes (Supplemental Table 3). These results indicate that *DRAIR* regulates some genes via direct interaction with chromatin — and others possibly through different mechanisms such as interaction with chromatin-modifying proteins.

**Figure 10:**
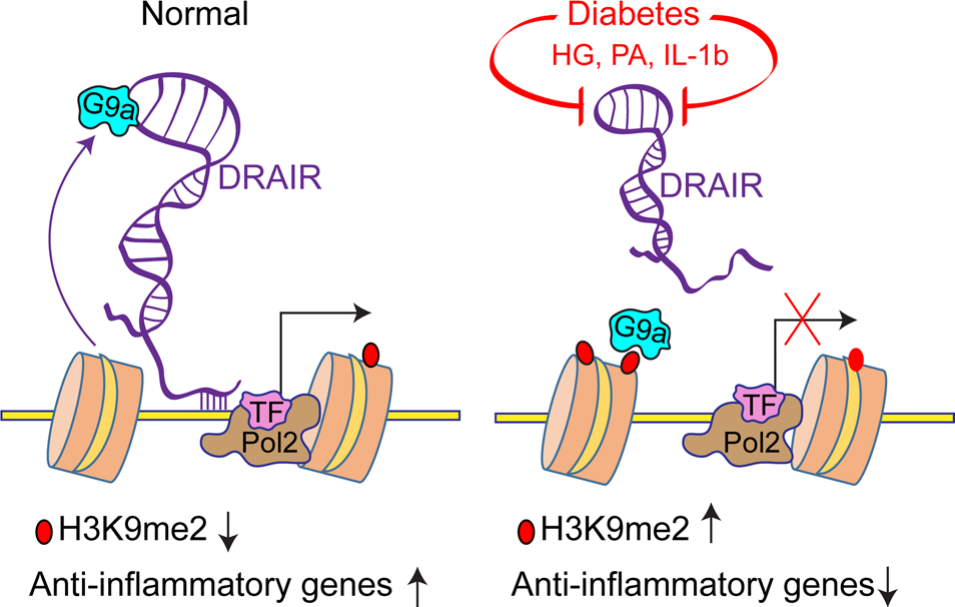
Schematic of DRAIR dysregulation in diabetes and epigenetic mechanisms of DRAIR actions. Under normal physiological conditions, lncRNA *DRAIR* reduces enrichment of repressive histone modifications, such as H3K9me2, at key antiinflammatory genes, by preventing recruitment of histone methyltransferase G9a (EHMT2), which allows the normal expression of these antiinflammatory genes. However, under diabetic conditions, downregulation of *DRAIR* and upregulation of G9a reverses these events, leading to repression of antiinflammatory genes, activation of monocytes, and chronic inflammation.

Accordingly, we found that interaction of *DRAIR* with the H3K9me2-methyltransferase G9a can regulate key target antiinflammatory genes. G9a can regulate gene expression via targeting transcriptionally active chromatin and interaction with lncRNAs such as *Kcnq1ot1* in cancer (42, 43). Previous studies showed dysregulation of H3K9me2 in diabetes (35), but the role of G9a has not been explored. Interestingly, our RNA-seq data show that G9a expression is increased in T2D monocytes, and treatment of THP1 monocytes with HG and PA also increased G9a protein levels, indicating a potential role for G9a in monocyte functions in T2D. We found that *DRAIR* reduces promoter enrichment of G9a and the corresponding repressive mark H3K9me2, along with upregulation of antiinflammatory genes such as *IL1RN* and *CPEB2*. This was further supported by our observation that *EHMT2* (G9a) knockdown could increase the expression of antiinflammatory genes that are also regulated by *DRAIR*. Therefore, *DRAIR* downregulation and G9a upregulation in T2D may act cooperatively in mechanisms associated with downregulation of antiinflammatory pathways in monocytes and chronic inflammation (Figure 10). However, *DRAIR* may also operate via G9a-independent mechanisms — such as regulation of H3K27me3, as in the case of *FCGR3B —* but further studies are needed to examine the role of H3K27me3 in *DRAIR* functions.

Our study also has limitations. The T2D subjects evaluated were relatively young; hence, it is unclear if *DRAIR* is also dysregulated in more longstanding T2D. In addition, we could not determine whether overexpression/reconstitution of *Drair* in *db/db* mice or other mouse models of T2D can attenuate inflammation or metabolic parameters because the technology to overexpress nuclear lncRNAs in vivo is not well developed. In addition, *Drair* knockdown did not inhibit *Il1rn* expression in mice PMs, instead showing a slight but not significant increase. These results suggest either a feedback response to increase inflammation or species-specific regulation. However, most importantly, *DRAIR* knockdown promoted inflammatory phenotype in both mice and humans, supporting its conserved antiinflammatory functions. But further work is needed to determine if the mouse and human orthologs operate through similar molecular mechanisms. We are also aware that genetic variations affecting the expression and function of lncRNAs can be associated with cardiometabolic disease (44, 45). Therefore, we searched *DRAIR* locus for genetic variants using The Cardiovascular Disease Knowledge Portal (https://cvd.hugeamp.org). However, we did not find any single nucleotide polymorphisms in the *DRAIR* locus that have significant association with human cardiovascular disease.

In summary, our results derived from multiple complementary approaches demonstrate that *DRAIR* regulates target gene expression in monocytes/macrophages via molecular and epigenetic mechanisms including direct interaction with chromatin and binding to key chromatin modifying proteins (Figure 10). Our findings emphasize the emerging role of antiinflammatory lncRNAs in metabolic diseases acting via RNA binding proteins and epigenetic mechanisms (23). Further understanding of such endogenous protective factors could aid in the development of much-needed therapies to ameliorate chronic inflammation and T2D complications.

## Methods

### Human CD14^+^ monocytes and THP1 monocytic cell line.

Fasting blood (15 mL) was collected from T2D and control volunteers (Supplemental Table 1). PBMCs from these samples were isolated and CD14^+^ monocytes purified by negative selection using magnetic beads (Miltenyi Biotech) as described (21). RNA from these samples were used for RNA-seq analysis and qPCR validation at City of Hope. For some in vitro experiments, human CD14^+^ monocytes from healthy volunteers were obtained from All Cells. Blood samples collected at City of Hope were used for all transfection experiments with human monocytes. The CD14^+^ monocytes from Ficoll-purified PBMC were isolated by immunomagnetic negative selection using EasySep Human Monocyte Isolation Kit (catalog 19059, Stemcell Technologies). Human THP1 monocytic cell line (American Type Culture Collection [ATCC]) was used to characterize *DRAIR* functions and mechanisms of actions. THP1 cells were cultured in RPMI containing 10% FBS, penicillin/streptomycin (Pen/Strp; 100 U/100 µg per mL), 2 mM glutamine, 5.5 mM glucose, and 50 μM β-mercaptoethanol. CD14^+^ monocytes were cultured in same medium without β-mercaptoethanol. Where indicated, monocytes were treated with NG for 72 hours (5.5 mM), HG (25 mM glucose) for 72 hours, PA (200 μM) for 24 hours, and HG + PA (PA added 48 hours after culturing in HG). Cells were then lysed in QIAzol (Qiagen) for RNA extraction. Human primary monocytes were also differentiated into macrophages using M-CSF1 (25 ng/mL) for up to 1 week, while THP1 cells were differentiated using PMA (20 ng/mL, up to 48 hours) and treated as indicated.

### Isolation of mice PMs.

Male *db/db* mice model of T2D (BKS.Cg-m^+/+^lepr^db^/J, catalog 00642), nondiabetic control *db/+* mice, and C57BL/6 mice were obtained from the Jackson Laboratory. Thioglycolate-elicited PMs were isolated from 10- to 12-week-old *db/db* and *db/+* mice and C57BL/6 mice as described (21, 22). PMs were plated in RPMI supplemented with 11 mM glucose and Pen/Strp for 1 hour, washed 3× with PBS, and RNA extracted. Mouse macrophage cell line RAW264.7 (ATCC, TIB-71) was cultured as described (21).

### RNA extraction and gene expression analysis.

RNA was isolated using RNeasy mini kit with on-column DNase I digestion (Qiagen). Reverse transcription followed by qPCR was performed by preparing cDNAs with Prime Script RT Master Mix (Takarabio) or QuantiTect RT-Kit (Qiagen), and qPCR with SYBR Green reagent using indicated gene primers (Supplemental Table 4) on a 7500 Fast Real-Time PCR system (Thermo Fisher Scientific). Data were analyzed using the 2^–ΔΔCt^ method to determine relative gene expression after normalization with internal control genes (*RPLPO, HPRT1,* and *PPIA* for human) and (*Rplp0* and *Ppia* for mouse genes) (21, 22).

### RNA-seq analysis.

Total RNA from CD14^+^ monocytes of T2D and control subjects (*n* = 5 each) was subjected to strand-specific RNA-seq on the Hi-Seq 2500 platform (Illumina). The data were analyzed using publicly available bioinformatics tools as described (21, 22). Raw sequences were aligned to the human reference genome hg19 using TopHat. RefSeq gene counts were normalized by trimmed mean of M value (TMM) method. DEGs were identified using Bioconductor package edgeR using criteria of fold change ≥ 2, FDR < 0.05, and average coverage ≥ 1 in at least 1 sample. DEGs were further analyzed by DAVID Functional Annotation Tool, IPA (Qiagen), and TRAP to identify GOs, significantly enriched pathways, and enrichment of TF binding sites in the promoters, respectively. The lncRNAs lacking coding potential were identified as described earlier (21, 22). Enrichment of biological processes in nearby DEGs were analyzed using Enrichr web server (46). Mouse orthologous Drair was identified using liftOver tools available in UCSC browser (https://genome.ucsc.edu/cgi-bin/hgLiftOver), which converts genome coordinates and genome annotation files between assemblies. RNA-seq data are available through the NCBI’s Gene Expression Omnibus (GEO) database (GSE156122; https://www.ncbi.nlm.nih.gov/geo/query/acc.cgi?acc=GSE156122).

### In vitro transcription/translation assay of DRAIR.

*DRAIR* cDNA was cloned into pcDNA3.1(+), and the resultant construct pDRAIR (Supplemental Figure 3B) was linearized by digestion with XhoI and subjected to in vitro transcription translation using the T7 TNT quick coupled transcription/translation system (Promega, catalog L1170). In parallel reactions, LUC transcript (positive control) and NTCs (negative control) were assayed. The reaction products were analyzed by Western blotting, and proteins were detected with Transcend colorimetric nonradioactive translation detection system (Promega) (22).

### Transfection of plasmids and oligonucleotides.

We used Dicer-substrate siRNAs (DsiRNAs) (Integrated DNA Technologies) and pooled siRNAs (Dharmacon) to knock down human *DRAIR* and *EHMT2* (G9a), respectively, and we used LNA-modified GapmeRs (Qiagen) to target mouse *Drair*. THP1 monocytes, THP-1 differentiated into macrophages (PMA, 20 ng/mL, 24–48 hours), human CD14^+^ monocytes differentiated into macrophages (M-CSF, 25–50 ng/mL for 7 days), and mouse RAW macrophages were transfected with indicated DsiRNs or siRNAs (20 nM) or GapmeRs (50-100 nM) using RNAiMAX (Thermo Fisher Scientific) or TransIT-KO (Mirusbio) following manufacturers’ protocols. Cells were used 48–72 hours after transfection for downstream analyses.

To overexpress *DRAIR*, freshly isolated CD14^+^ monocytes were transfected with pDRAIR or control pcDNA3.1 (EV) using Human Monocyte Nucleofector Kit (Lonza) on a Nucleofector device II and program Y-001. The next day, transfected cells were treated ± LPS (100 ng/mL) for 24 hours, and RNA were isolated. THP1 cells were also transfected with pDRAIR or control pcDNA3.1 (EV) with TransIT-2020 (Mirusbio), and stably transfected cells were selected using G418 (400 μg/mL). In some experiments, *DRAIR* cDNA was cloned into lentiviral vector pLentipuro (Addgene). Lentiviruses expressing *DRAIR* or control *EGFP* were prepared using cotransfection with helper plasmids in HEK293T cells. THP1 cells were transduced with *DRAIR* and *EGFP* lentiviruses using polybrene (MilliporeSigma, 8 μg/mL) overnight and used a week after transduction.

Mouse *Drair* knockdown in vivo. Male C57BL/6 mice (8 weeks old) were injected (i.p.) with thioglycolate (3%), followed by i.p. injection of in vivo grade *Drair* Gapmer or negative control Gapmer (Qiagen) at 24 and 72 hours (5 mg/kg). PMs were isolated at 96 hours for gene expression analysis.

### Cloning of DRAIR promoter and transfection of reporter plasmids.

Human *DRAIR* promoter (–1064 to +39) was PCR amplified from genomic DNA and subcloned upstream of the firefly LUC gene to generate pDRluc. Transcription factor binding sites enriched in *DRAIR* promoter were analyzed using TRANSFAC software (Qiagen). THP1 cells were cotransfected with pDRluc, internal control SV40-Renilla LUC, and indicated plasmids using TransIT-2020. The following day, treatments were as indicated for 24 hours, and LUC activities in cell lysates were determined using Dual-LUC Reporter Assay System in GloMax Luminometer (Promega). Firefly/Renilla ratios were reported as fold over controls.

### Phagocytosis assays.

THP1 cells differentiated into macrophages were transfected with siDR or siNC and treated as indicated. The next day, phagocytosis assays were performed in 96-well black plates with clear flat bottoms using FITC-labeled *E*. *coli* BioParticles as described by the manufacturer (Vybrant Phagocytosis Assay Kit, Thermo Fisher Scientific). Fluorescence from phagocytosed *E.coli* bioparticles was measured at 483/518 nm on an Infinite 200Pro plate reader (Tecan), and results were reported as arbitrary fluorescence units.

### RNA isolation from nuclear, cytoplasmic, and chromatin fractions.

RNA from nuclear and cytosolic fractions was purified using columns and protocols supplied with PARIS kit (Thermo Fisher Scientific). RNA fractions from chromatin and soluble nuclear extracts were isolated as described (22, 23). Levels of indicated transcripts levels were determined by qPCR.

### RNA-FISH.

RNA-FISH was performed with LNA-Cy5–labeled oligonucleotide probes targeting *DRAIR* (EXIQON-QIAGEN) as described (22). Images were captured and processed using a Zeiss Observer Z1 wide field microscope and ZEN Blue software (Zeiss).

### Western blot analysis.

THP1 cells were treated as indicated with NG, HG, PA, and HP and centrifuged at 100*g* for 10 minutes at 4°C. Cell pellets were washed with cold PBS and lysed in Nuclear Isolation Buffer (NEB) containing 0.25M sucrose, 8 mM Tris HCl (pH 7.4), 5 mM MgCl_2_, 0.8% Triton X-100, and 1× Complete Protease inhibitor (Roche) on a rotator for 20 minutes at 4°C. Cell lysates were centrifuged at 2500*g* for 20 minutes at 4°C; nuclear pellets were lysed in Laemmli sample buffer (without dye and β-mercaptoethanol) and were briefly sonicated (15 seconds at 4°C; Diagenode) to reduce viscosity. Protein concentrations were estimated using Protein Assay kit (Bio-Rad), and equal amounts of nuclear proteins were subjected to Western blotting with G9a antibody (1:1000, catalog 688851S, Cell Signaling Technology) and internal control Histone H3 antibody (1:3000, catalog ab1791, Abcam) (22). Intensities of protein bands detected by enhanced chemiluminescence were quantified using a GS-900 calibrated densitometer and Image Lab software (Bio-Rad). Results were expressed as the ratio of G9a/H3.

### ChIRP.

*DRAIR* interaction with chromatin and with nuclear proteins were determined using ChIRP assays and ChIRP-MS, respectively (47, 48). For ChIRP assays,THP1 cells (80 million) were fixed with glutaraldehyde for 30 minutes, washed, and lysed in ChIRP lysis buffer containing SUPERase-in RNA inhibitor (Thermo Fisher Scientific) and Complete protease inhibitors (Roche). Lysates were sonicated in Bioruptor to fragment DNA (200–1000 bp) and diluted with hybridization buffer (1:2). Then, DRAIR tiling biotinylated oligonucleotides (Stellaris) were added (10 pg/mL) and incubated overnight at 37°C. The following day, ChIRP complexes were captured using streptavidin-magnetic beads and washed 5 times, and ChIRP-RNA and ChIRP-DNA were eluted from the beads. ChIRP-RNA was used to estimate *DRAIR* recovery. ChIRP-DNA was used for DNA-seq on Illumina platform (HiSeq 2500). RAW reads were aligned to the human hg19 reference genome, and Dbs on chromatin identified as described (47). ChIRP-seq data are available through the NCBI’s GEO database (GSE156122). ChIRP-DNA was also analyzed by ChIRP-qPCR to validate Dbs with specific primers.

To identify *DRAIR*-interacting proteins, THP1 cells stably overexpressing *DRAIR* (400 million cells) were fixed with formaldehyde (3%) for 30 minutes (48), and cell lysates were processed as described for ChIRP. Biotinylated oligonucleotides targeting LUC transcript were used as negative controls. Nucleic acid–protein complexes were captured using streptavidin-magnetic beads and boiled in SDS-Laemmli protein sample buffer, and eluted proteins were fractionated on precast SDS-PAGE (4%–15%) gels (Bio-Rad). Gels were stained with SimplyBlue SafeStain, and different regions from each lane (*DRAIR* and *LUC* probes) were subjected to MS at City of Hope’s Mass Spectrometry Core as described (22, 23). Differentially interacting proteins with *DRAIR* versus LUC probes were analyzed using Scaffold 3.0. *DRAIR*-interacting proteins were further analyzed by STRING database to identify enrichment of biological processes (34) and were validated using RNA pulldown and RIP assays.

### RIP assays.

THP1 cells were fixed with formaldehyde (1%) for 10 minutes at room temperature, washed with ice cold PBS, lysed in nuclear isolation buffer supplemented with RNase and protease inhibitors, and centrifuged at 2500*g* for 20 minutes at 4°C to collect the nuclear pellet. Nuclei were lysed in RIPA buffer, sonicated, and immunoprecipitated overnight at 4°C with G9a antibody (5 μg, catalog 68851S), SUV39H1 antibody (5 μg, catalog 8729S), or negative control IgG (catalog 2729S) (all from Cell Signaling Technology). Immune complexes were collected on IgG-magnetic beads, washed 5 times with RIPA buffer, and incubated in elution buffer containing proteinase K at 55°C for 30 minutes. RNA from supernatants was purified using RNeasy MinElute Cleanup Kit (Qiagen) and analyzed by qPCR.

### RNA pulldown assays.

The pcDNA3.1(+) pDRAIR and pcDNA3.1(–) expressing DRAIR anti-sense (pDRAIR-AS, Supplemental Figure 3C) were linearized to prepare biotinylated DRAIR and DRAIR-AS probes, respectively, by in vitro transcription with T7-RNA polymerase using Biotin RNA Labeling Mix kit (Roche). Nuclear extracts from THP1 cells (1 mg protein) were incubated with 1 μg of biotinylated probes, and RNA pulldown assays were performed as described (22). Proteins eluted from biotinylated RNA-protein complexes were subjected to immunoblotting with G9a antibody (1:1000). Protein bands were detected using Enhanced Chemiluminescence kit (Perkin Elmer).

### ChIP.

ChIP assays were performed as described previously (22, 49), with some modifications. THP1 cells crosslinked with 1% formaldehyde were lysed in nuclear isolation buffer (20 minutes, 4°C) and centrifuged (2500*g*, 20 minutes) at 4°C. Nuclear pellets were lysed in ChIP lysis buffer, and chromatin was sheared by sonication using a Bioruptor. Sonicated nuclear lysates were diluted 1:10, and lysates containing equal amounts of DNA were immunoprecipitated using antibodies against H3K9me2 (catalog 4658S), G9a (catalog 68851S), or H3K27me3 (catalog 9733S) obtained from Cell Signaling Technology, or KLF4 (10 μg, catalog AF3640, R&D Systems). Immunocomplexes were captured using IgG magnetic beads, washed, and eluted as indicated (22, 49), except that high salt wash was performed 2 times. ChIP DNA was reverse crosslinked overnight at 67°C and treated with RNAse A (10 μg/mL) for 30 minutes at 37°C. DNA was extracted using QIAquick PCR Purification Kit. ChIP-DNA was analyzed in triplicate by ChIP-qPCR using indicated primers and SYBR Green reagent on the 7500 Fast Real-Time PCR system. The ChIP-qPCR data were analyzed using the formula 2^–(CtChIP-Ct100%input)^, and results were normalized with input were expressed as percentage of input.

### THP1 monocyte–EC binding assays.

THP1 monocytes transfected with siDR or siNC were treated with or without TNF-α for 5 hours (10 ng/mL), labeled with DAPI (5 ng/mL, 20 minutes), and washed twice with PBS. Then, labeled monocytes (125,000 per cm^2^) were incubated with confluent HUVECs in 24-well plates in serum-depletion medium (MCDB-131 [MilliporeSigma] containing 2.5% FBS and 1× antibiotic/antimycotic agents) for 30 minutes at 37°C. Nonadherent monocytes were removed by washing twice with PBS. EC-bound monocytes were fixed with 1% paraformaldehyde for 15 minutes and washed, and images from multiple wells/group were captured using a fluorescence microscope (×100 magnification). Bound monocytes were counted with ImageJ software (NIH), and results are expressed as monocytes/field (mean ± SD).

### Statistics.

Graphpad Prism (7.0 and above) software was used to perform statistical analysis. Data are represented as mean ± SD of experiments performed at least in triplicate. Shapiro-Wilk normality test was used to test normal distribution of each sample group. Comparisons between 2 groups were performed using 2-tailed unpaired *t* tests. Comparison among more than 2 groups were performed using 1- or 2-way ANOVA, followed by multiple-comparison tests as indicated in the figure legends. *P* < 0.05 was considered statistically significant.

### Study approval.

All human blood samples were collected after written informed consent from T2D and control volunteers. The study was approved by the IRBs at City of Hope and Baylor College of Medicine. Mouse studies were approved by IACUC at City of Hope and conducted in accordance with the *Guide for the Care and Use of Laboratory Animals* (National Academies Press, 2011).

## Author contributions

MAR conceptualized the work, designed and performed most experiments, and wrote the manuscript. VA, RG, MW, LL, LZ, and MA performed experiments and analyzed data. XW, ZC, and VST performed bioinformatics analysis. S. Das performed experiments, assisted with experimental design, and edited the manuscript. S. Devaraj provided human monocytes for RNA-seq analysis. RN conceptualized the work, edited the manuscript, acquired funding, and supervised the study. RN and MAR are the guarantors of this work and, as such, had full access to all the data in the study and take responsibility for the integrity of the data and the accuracy of the data analysis.

## Supplementary Material

Supplemental data

## Version 1 05/04/2021

In-Press Preview

## Version 2 06/08/2021

Electronic publication
