# Supplementary material for: lncRNA DRAIR is downregulated in diabetic monocytes and modulates the inflammatory phenotype via epigenetic mechanisms
Source: JCI Insight. 2021 Jun 8;6(11):e143289. doi: 10.1172/jci.insight.143289 (PMC8262346; doi:10.1172/jci.insight.143289)
Supplement: Supplemental data [file jciinsight-6-143289-s225.pdf]

Supplementary Material

LncRNA *DRAIR* is downregulated in diabetic monocytes and modulates inflammatory phenotype via epigenetic mechanisms

Marpadga A. Reddy, Vishnu Amaram, Sadhan Das, Vinay Singh Tanwar, Rituparna Ganguly, Mei Wang, Linda Lanting, Lingxiao Zhang, Maryam Abdollahi, Zhuo Chen, Xiwei Wu, Sridevi Devaraj and Rama Natarajan

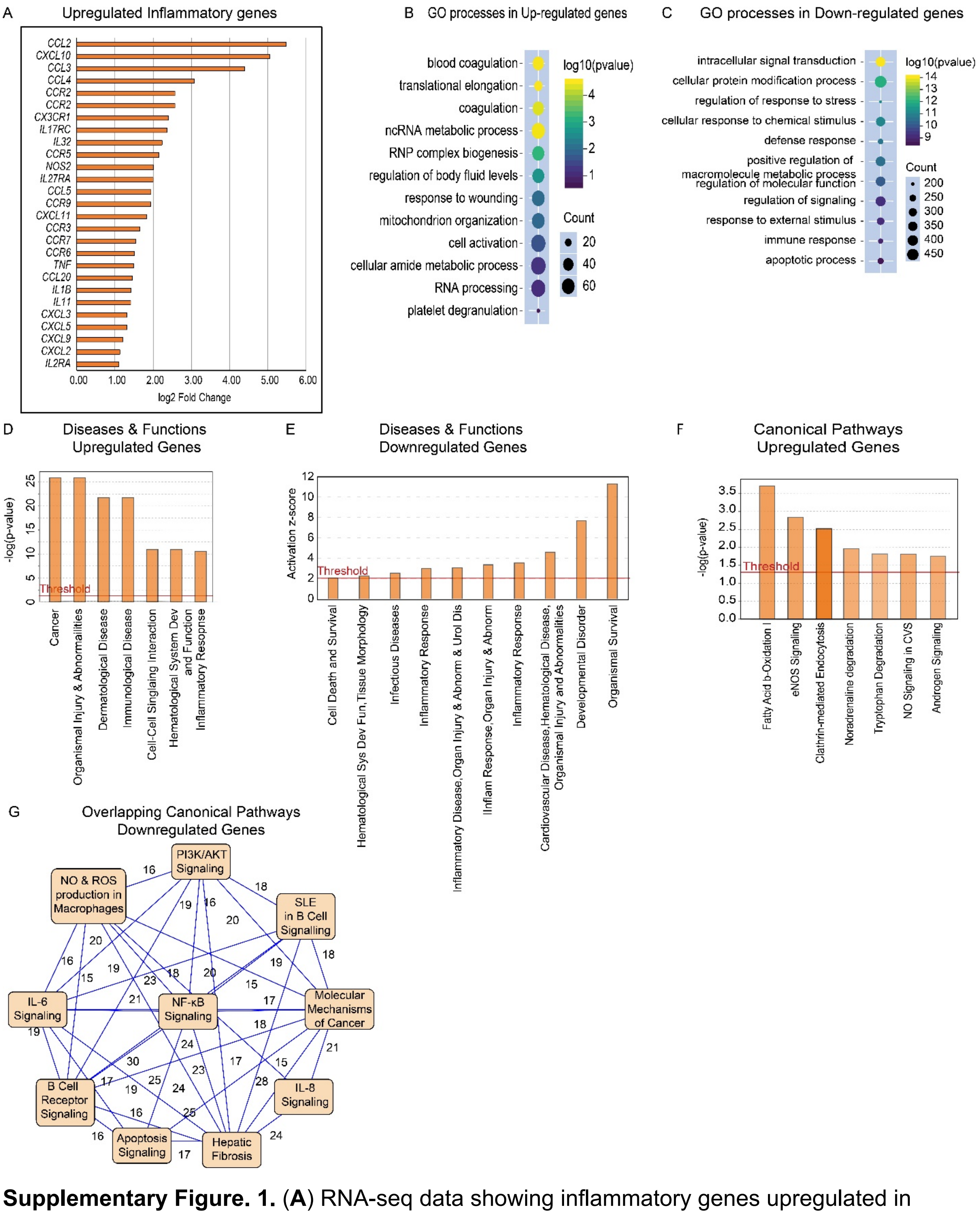

Supplementary Figure. 1. (**A**) RNA-seq data showing inflammatory genes upregulated in monocytes from humans with type 2 diabetes versus controls. (**B-C**) Bubble plots depicting GO Processes enriched in upregulated and downregulated genes. Bubble color represents significance (p-values), and bubble size indicates gene count. (**D-E**) IPA analysis showing Diseases & Functions enriched in upregulated (D) and downregulated genes (E). (**F**) IPA analysis showing Canonical pathways enriched in upregulated genes. (**G**) IPA analysis showing

Overlapping Canonical pathways in downregulated genes. Numbers indicate common genes in overlapping networks. SLE: Systemic Lupus Erythematosus. In panels D and F, y-axis represents –log(p-values) from Fisher's exact test. In panel E, y-axis shows Z-score, where 2 is set as threshold.

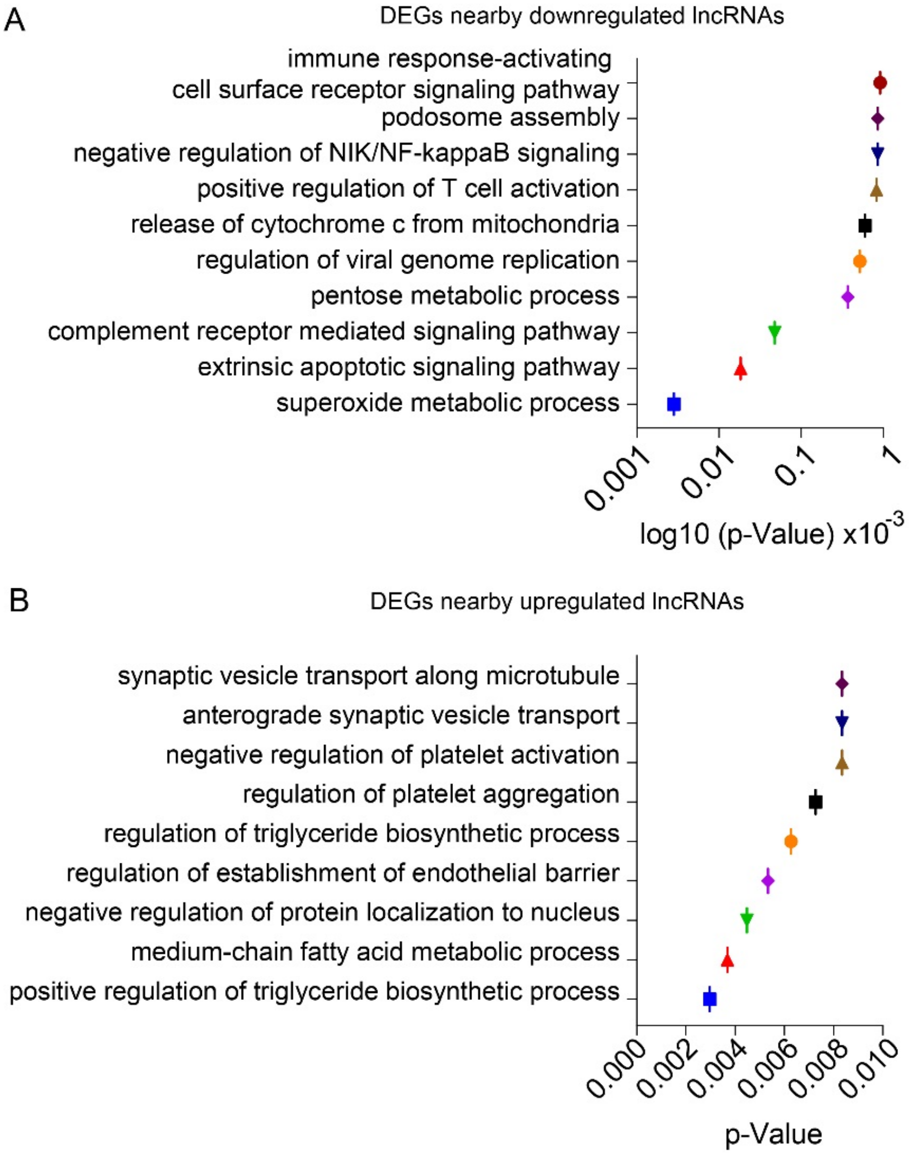

Supplementary Figure. 2. (A-B) GO Biological processes enriched among differentially expressed gene (DEG)s located nearby the downregulated (**A**) and upregulated (**B**) lncRNAs in T2D. DEGs nearby differentially expressed lncRNAs (± 250 kb) in T2D were analyzed using the web-based gene list enrichment analysis tool Enrichr (1-2).

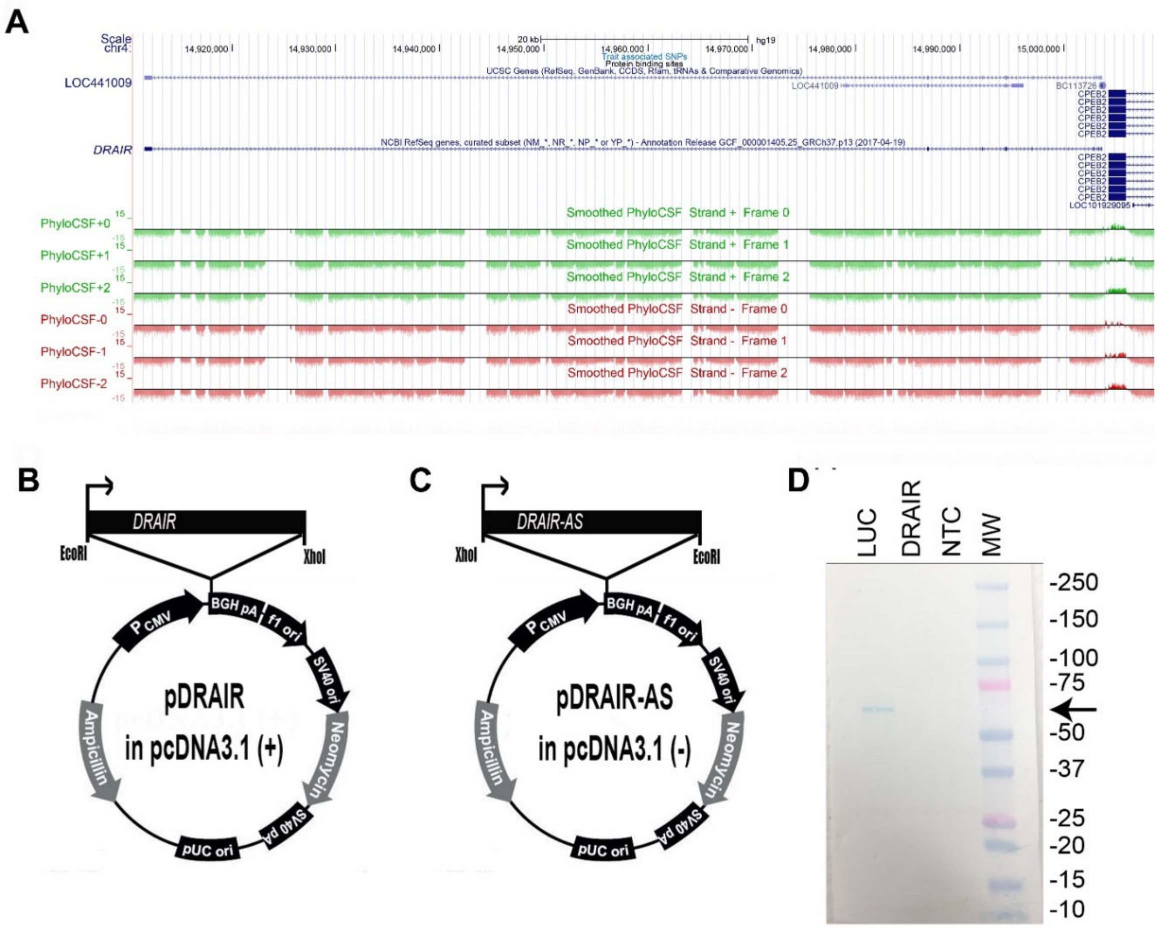

Supplementary Figure 3. Characterization of *DRAIR* coding potential. (**A**) The Raw PhyloCSF tracks of *DRAIR* genomic regions, showing the PhyloCSF score of less than zero in all six reading frames, predicting that DRAIR lacks coding potential. In addition, Coding Potential Calculator 2 (CPC2) software (3) also predicted that *DRAIR* lacks coding potential (coding probability 0.0302545). (**B, C**) Plasmid maps of pDRAIR expressing *DRAIR* (**B**) and pDRAIR-AS expressing *DRAIR* in antisense orientation (**C**). DRAIR cDNA was cloned downstream of the CMV promoter into EcoR1 and XhoI sites of pcDNA3.1 (+) and pcDNA3.1 (-) plasmids to generate pDRAIR and pDRAIR-AS vectors respectively. Arrow indicates direction of transcription. (**D**) Western blot of *in vitro* translation products derived from luciferase (LUC) RNA (positive control), DRAIR RNA, and no template control (NTC) reactions. pDRAIR was used as a template in an *in vitro* coupled transcription-translation system, and reactions were analyzed by Western blot. Protein products were visualized using colorimetric nonradioactive detection system (Transcend Non-Radioactive Translation Detection Systems, Promega) that detects biotinylated lysines. Arrow indicates 62 kD luciferase protein. MW: Pre-stained protein molecular weight markers.

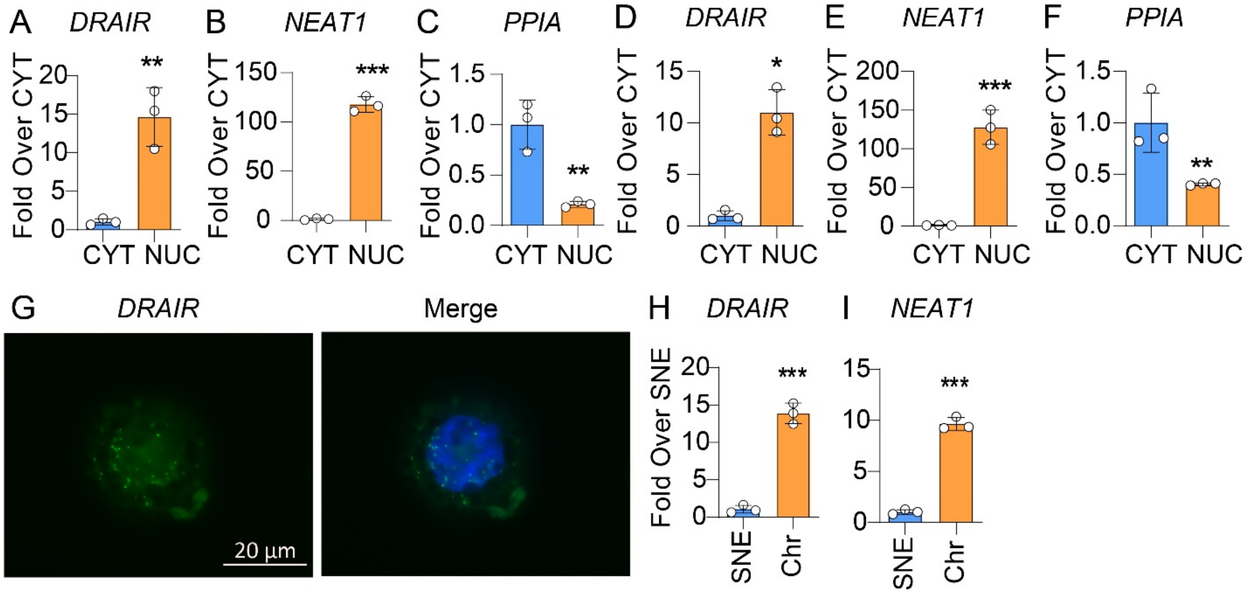

Supplementary Figure 4. DRAIR is a nuclear lncRNA and is enriched in chromatin.(**A-F**) RT-qPCR analysis of *DRAIR* expression in cytoplasmic (CYT) and nuclear (NUC) fractions from THP1 monocytes (**A-C**) and THP1 monocytes converted to macrophages with PMA (**D-F**). (**G**) Images showing RNA-FISH analysis of *DRAIR* localization in THP1 macrophages. Green spots: *DRAIR* probe; Blue-DAPI staining of nuclei. (**H and I**) RT-qPCR analysis of indicated transcripts in soluble nuclear extracts (SNE) and chromatin (Chr) fractions from THP1 cells (n=3). *, p<0.05; **, p<0.01; ***, p<0.001 as determined by Student's t-test (n=3).

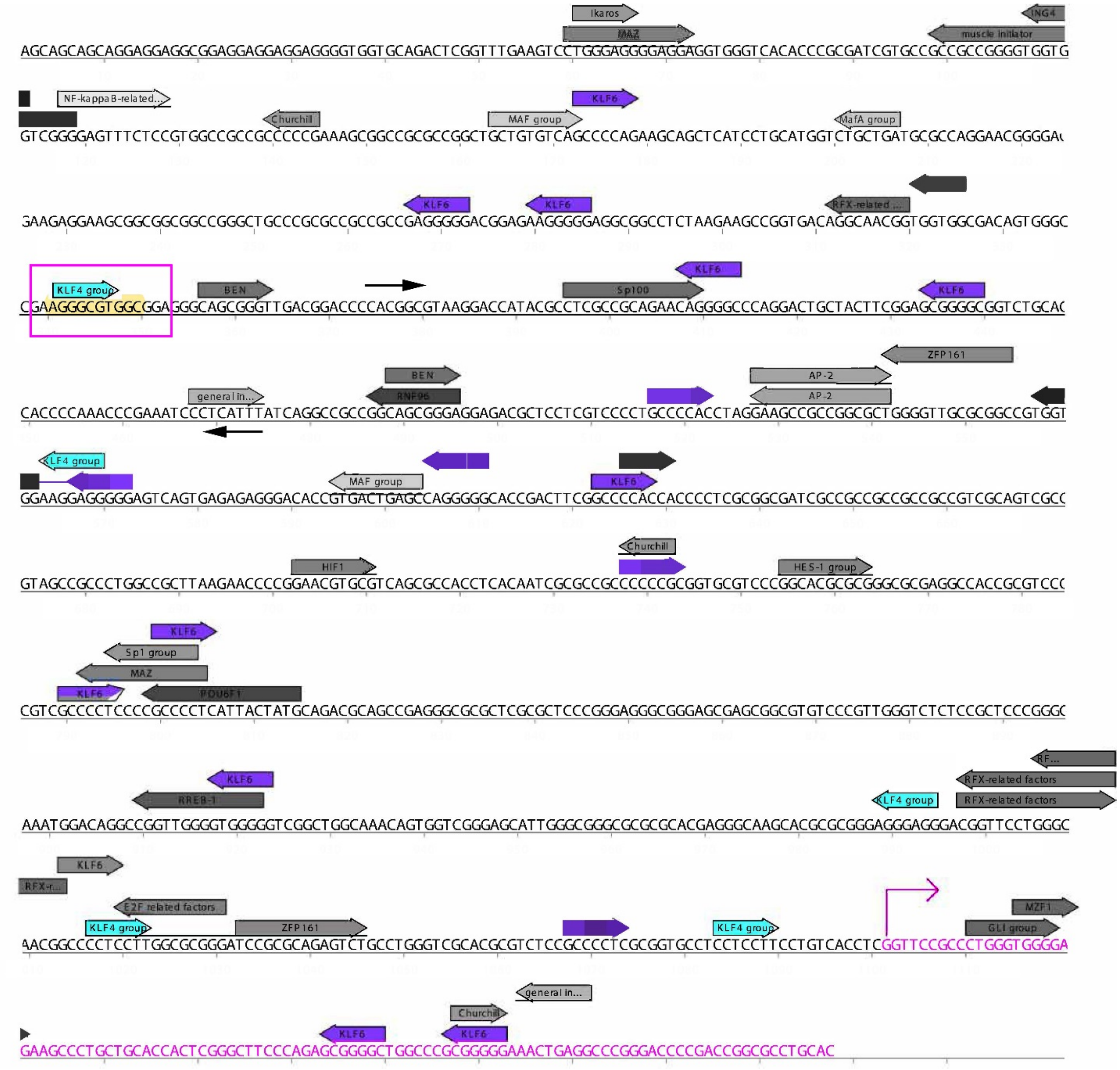

Supplementary Figure 5. Results of Transfac analysis of *DRAIR* promoter showing potential binding sites for transcription factors. KLF4 motifs are indicated by light blue boxes and KLF6 motifs by purple boxes. Direction of the arrows indicates strand orientation. DRAIR transcript start site is indicated by a pink color arrow. Potential KLF4 binding site validated by ChIP assays is shown by pink rectangle. Black arrows indicate location of ChIP primers.

Factor Name	Adjusted p-Value	Enriched Motiff Consensus sequence
RBP-Jkappa	5.52E-03	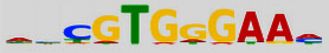
E2A group	8.76E-03	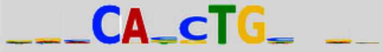
muscle initiator	6.10E-03	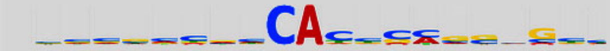
RREB-1	8.73E-03	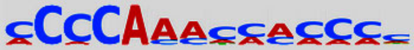
Hox9-12	8.73E-03	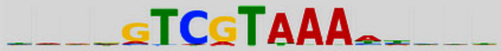
FPM315	5.86E-03	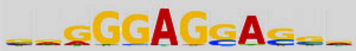
NF-1 factors	5.47E-03	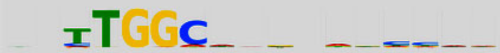
AP-2	1.52E-05	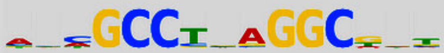
ZFP161	3.07E-05	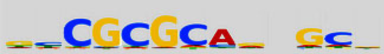
RNF96	4.29E-03	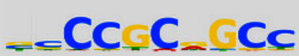
BEN	6.18E-04	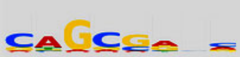
KLF6	2.12E-03	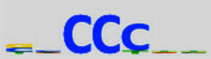

Supplementary Figure 6. Transcription factor motifs in enriched in *DRAIR* binding sites identified with ChIRP-seq analysis using *DRAIR* probes in THP1 cells. TF motifs enriched in 152 genomic regions containing DRAIR binding sites were analyzed with Transfac software using default parameters. Consensus sequences and adjusted p-values of the top 12 TF enriched motifs are shown.

A DRAIR Interacting proteins

Accession	Name	Function
Histone methyltransferases/histone demethylases		
NP_001276342.1	EHMT2a/G9a	H3K9me1/2
NP_001140160.1	JMJD1A/ KDM3A	H3K9me1/2
NP_001269877.1	JMJD1C/KDM3C	H3K9me1/2
NP_001269095.1	SUV39H1	H3K9me3
NP_060834.2	RSBN1/KDM9	??
NP_001184033.1	MLL1/KMT2A	H3K4me3
XP_024306267.1	SETD1A/KMT2F	H3K4me3
NP_001164100.1	CHD8	MLL1 complex<br>TP53 complex
NP_001073315.1	CBP	HAT
NP_001294936.1	EED	PRC
Meditaor Proteins		
NP_057063.2	MED23	SP1/TR/Vit D
NP_004220.2	MED14	SP1/TR/Vit D
NP_001254726.1	MED24	SP1/TR/Vit D
DNAme bind proteins		
NP_001104262.1	MECP2	CpG
RNA methylation		
XP_011530464.1	METTL14	N6A-MT
NP_060228.3	ALKBH5	m6A-demethylase
NP_055865.1	CMTR1	Cap MT
Transcription Repressors		
NP_057368.3	CNOT1	Repressor
NP_001258563.1	CNOT9	Repressor
NP_001272758.1	CEBPA	Tum Supp

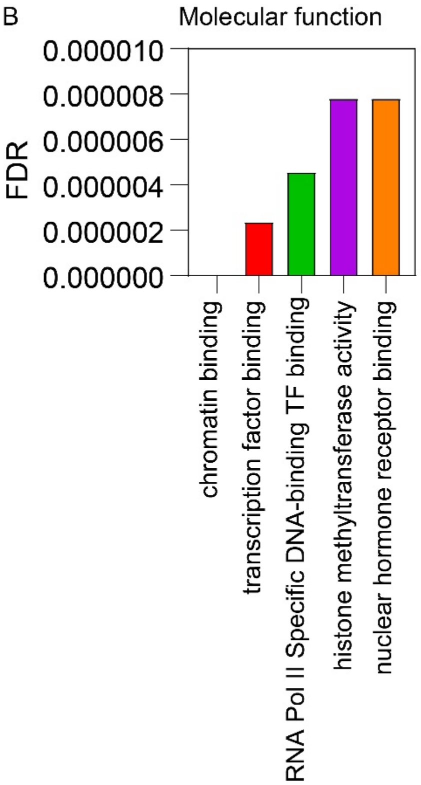

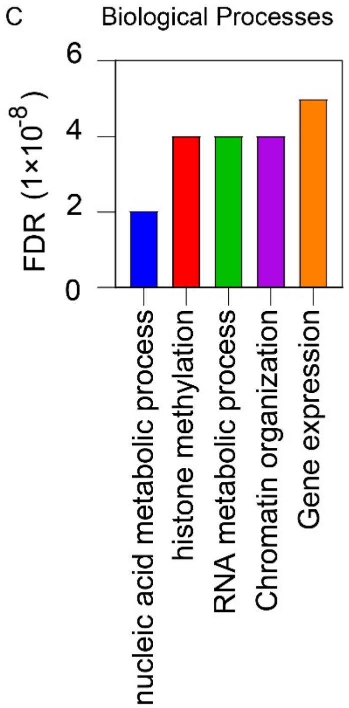

Supplementary Figure 7. *DRAIR* interacting proteins identified by ChIRP-mass spectrometry (ChIRP-MS). (**A**) Subset of *DRAIR* interacting proteins identified in ChIRP-MS. THP1 cells overexpressing *DRAIR* were used to perform ChIRP assays. Cell lysates were incubated with biotinylated DRAIR probes or luciferase probes (negative control). After overnight incubation, nucleic acid-protein complexes were captured on streptavidin-beads, washed to remove non-specific interactions, and eluted with SDS-sample buffer. Eluted samples were fractionated on 4-15% SDS-polyacrylamide gels and stained with Coomassie blue. Proteins pulled down by DRAIR or LUC probes, and located between the 250 and 20 kDa molecular weight markers were analyzed by mass spectrometry (MS) to identify *DRAIR* interacting proteins. (**B, C**) GO Molecular functions and Biological processes enriched in *DRAIR* interacting proteins.

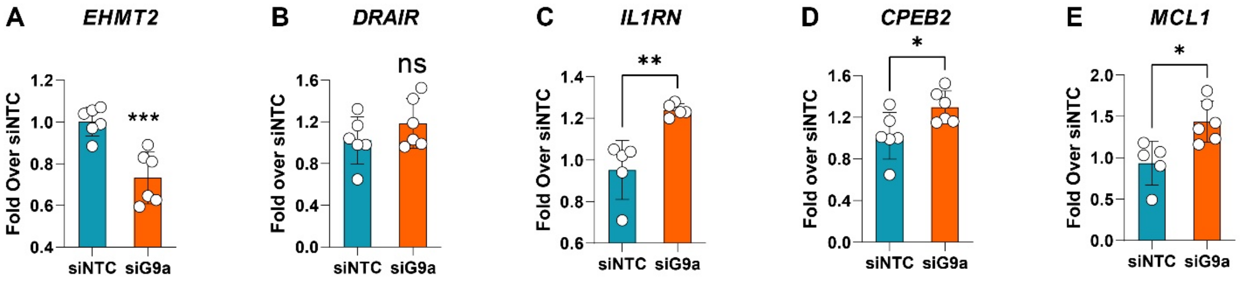

Supplementary Figure 8. EHMT2 (G9a) knockdown increases anti-inflammatory genes in THP1 cells. (**A-E**) RT-qPCR analysis of indicated genes in THP1 cells transfected with siRNAs targeting *EHMT2* (siG9a) or non-targintg control siRNA (sNTC) (n=5-6). *, p<0.05; **,p<0.01; ***,p<0.001 as determined by unpaired t-tests (n=6).

Supplementary Table 1. Characteristics of volunteers without (control) and with type 2 diabetes (T2D).

	**Control**	**T2D**
Age (yrs)	21.2 ± 8.2	21.2 ± 7.7
Sex	3 Male	3 Male
	2 Female	2 Female
Body mass index (Kg/m^2)	22.4 ± 2.1	21.4 ± 3.1
Blood glucose (mg/dL)	87.2 ± 9.2	190 ± 57.6**
Hemoglobin A1c(%)	5.02 ±0.11	9.32 ± 2.4**
Antibodies	Not determined	Not detected
C-reactive protein (mg/L)	Not determined	3.7 ± 0.6
C-peptide (ng/mL)	Not determined	5.7 ± 1.0

Mean ± SEM, **, p<0.005 vs Normal, n=5 each
To determine Type 2 diabetes, information about body weight and body mass index (BMI), blood levels of HbA1c, glucose, antibodies and C-peptide were measured. C-reactive protein was measured as a marker for inflammation in T2D.
T2D diagnosis was based on blood glucose on 2 occasions > 126 mg/dL; HbA1c > 6.5%, no antibodies, detectable C-peptide and sometimes obesity.
Family history, diabetes medication status and length of diabetes were not recorded.
Blood samples for monocyte preparation were collected once.

Supplementary Table 2. ChIRP Peaks using DRAIR probes (n=2, Hg19)		
**Chromosome**	**Start**	**End**
chr1	5790512	5790724
chr1	9637587	9637777
chr1	17425128	17425360
chr1	22489192	22489478
chr1	40498794	40499106
chr1	41310615	41310880
chr1	42444193	42444465
chr1	44729428	44729646
chr1	50442794	50443171
chr1	59106831	59107065
chr1	59462869	59463108
chr1	103520933	103521120
chr1	110469466	110469821
chr1	121484319	121485432
chr1	182129542	182129757
chr1	185469917	185470147
chr1	203471357	203471578
chr1	204828349	204828553
chr1	210016420	210016674
chr1	218351110	218351306
chr1	229241728	229241981
chr1	231147087	231147675
chr1	244598725	244598927
chr10	80574413	80574671
chr10	94968805	94969171
chr10	104537592	104537821
chr10	127846810	127847591
chr10	133881322	133881674
chr10	134756323	134756520
chr11	18779518	18779747
chr11	42530753	42530940
chr11	70217419	70217619
chr12	100516736	100516956
chr13	46521080	46521267
chr13	47695847	47696054
chr13	77303784	77303976
chr13	99786808	99787132
chr14	35991914	35992101
chr14	45033490	45033707
chr14	66170924	66171114
chr14	76533456	76533643
chr14	97059540	97059788
chr14	100847153	100847354

Supplementary Table 2. ChIRP Peaks using DRAIR probes (n=2, Hg19)		
**Chromosome**	**Start**	**End**
chr14	103816304	103816829
chr15	23051401	23051622
chr15	39376017	39376206
chr15	62664996	62665186
chr16	10175015	10175231
chr16	26824106	26825019
chr16	26825147	26825364
chr17	41352045	41352336
chr17	48586780	48586967
chr17	78903751	78904321
chr18	21979534	21979739
chr18	72092245	72092674
chr19	12370314	12370501
chr19	12512250	12512493
chr19	13119518	13119718
chr19	13811138	13811359
chr19	39627393	39627580
chr2	22031293	22031595
chr2	43182872	43183215
chr2	51330566	51330812
chr2	64924611	64924991
chr2	95704318	95704621
chr2	128183321	128183636
chr2	174103628	174103884
chr2	191435143	191435354
chr2	197816517	197816883
chr2	204399823	204400106
chr2	228430670	228430905
chr2	233403373	233403606
chr20	6699245	6699432
chr20	21653263	21653501
chr20	24894194	24894448
chr20	30311769	30312022
chr20	42572922	42573109
chr20	42647657	42647848
chr20	61484300	61484541
chr21	9826013	9826275
chr21	43772027	43772348
chr21	45942629	45945406
chr21	47478300	47478731
chr3	11246041	11246407
chr3	15501414	15501601
chr3	36777605	36777973

Supplementary Table 2. ChIRP Peaks using DRAIR probes (n=2, Hg19)		
Chromosome	Start	End
chr3	51422588	51422902
chr3	65884827	65885014
chr3	72387306	72387626
chr3	98697654	98697876
chr3	127805151	127805416
chr4	3108737	3109019
chr4	37476192	37476382
chr4	61082157	61082390
chr4	130717798	130717999
chr4	143340902	143341131
chr4	145622591	145622787
chr4	154576071	154576582
chr4	181174378	181174565
chr4	181960766	181960997
chr5	435133	435362
chr5	2211196	2211459
chr5	5274428	5274643
chr5	31671098	31671347
chr5	58909228	58909421
chr5	110520441	110520737
chr6	35681204	35681562
chr6	36823859	36824133
chr6	58776935	58777382
chr6	58777447	58777893
chr6	58778152	58779226
chr6	90241526	90241790
chr6	92211196	92211418
chr6	100725913	100726100
chr6	130421309	130421512
chr6	144621969	144622156
chr6	149765651	149765975
chr6	150239190	150239411
chr6	150385596	150385872
chr6	158441396	158441731
chr6	164039932	164040171
chr6	164348450	164348641
chr6	168069287	168069603
chr6	170810193	170810787
chr6	170811121	170811654
chr6	170811970	170812370
chr7	77022135	77022339
chr7	84793282	84793525
chr8	49060249	49060449

**Supplementary Table 2. ChIRP Peaks using DRAIR probes (n=2, Hg19)**		
**Chromosome**	**Start**	**End**
chr8	55243794	55244029
chr8	56517055	56517246
chr8	120423472	120423687
chr8	130775058	130775260
chr8	143266231	143266418
chr8	144722404	144722594
chr9	80506363	80506621
chr9	87879309	87880385
chr9	96698635	96699304
chr9	114700904	114701161
chr9	131564641	131564941
chr9	138752833	138753033
chrM	0	631
chrM	2670	2932
chrM	3080	3942
chrM	9682	10290
chrM	10672	10954
chrM	11851	12124
chrM	12317	12634
chrM	12733	13172
chrM	13596	14089
chrM	14147	14561
chrM	14772	16571

Supplementary Table 3. List of differentially expressed genes nearby *DRAIR* binding sites	
**Symbol**	**Entrez Gene Name**
IL1R2	interleukin 1 receptor type 2
MME	membrane metalloendopeptidase
ENTPD2	ectonucleoside triphosphate diphosphohydrolase 2
CBS/CBSL	cystathionine-beta-synthase
EPHB1	EPH receptor B1
CSF3R	colony stimulating factor 3 receptor
VNN2	vanin 2
ELL	elongation factor for RNA polymerase II
PLAUR	plasminogen activator, urokinase receptor
SBNO2	strawberry notch homolog 2
CDC34	cell division cycle 34
IL18RAP	interleukin 18 receptor accessory protein
ARHGEF17	Rho guanine nucleotide exchange factor 17
ANK1	ankyrin 1
CHSY1	chondroitin sulfate synthase 1
ECE1	endothelin converting enzyme 1
PDE2A	phosphodiesterase 2A
SLC11A1	solute carrier family 11 member 1
GAB2	GRB2 associated binding protein 2
LYN	LYN proto-oncogene, Src family tyrosine kinase
ARHGAP26	Rho GTPase activating protein 26
ULK1	unc-51 like autophagy activating kinase 1
CD82	CD82 molecule
PTPRE	protein tyrosine phosphatase, receptor type E
ASAP1	ArfGAP with SH3 domain, ankyrin repeat and PH domain 1
PREX1	phosphatidylinositol-3,4,5-trisphosphate dependent Rac exchange factor 1
NOTCH1	notch 1
CHD4	chromodomain helicase DNA binding protein 4
TFR2	transferrin receptor 2
ERMN	ermin
SPAG9	sperm associated antigen 9
PTP4A1	protein tyrosine phosphatase type IVA, member 1
MEF2D	myocyte enhancer factor 2D
PHF21A	PHD finger protein 21A
ARNTL	aryl hydrocarbon receptor nuclear translocator like
MLLT1	MLLT1, super elongation complex subunit
AGO2	argonaute 2, RISC catalytic component
NBEAL2	neurobeachin like 2
EGLN1	egl-9 family hypoxia inducible factor 1
TBL1X	transducin beta like 1X-linked
LASP1	LIM and SH3 protein 1
RAB7A	RAB7A, member RAS oncogene family
NFIC	nuclear factor I C
PKN2	protein kinase N2
CSF2RB	colony stimulating factor 2 receptor beta common subunit
ESPL1	extra spindle pole bodies like 1, separase
EP300	E1A binding protein p300
MED13L	mediator complex subunit 13 like
COL18A1	collagen type XVIII alpha 1 chain
NFKBIE	NFKB inhibitor epsilon
ABCC2	ATP binding cassette subfamily C member 2
DDAH2	dimethylarginine dimethylaminohydrolase 2
ARID4B	AT-rich interaction domain 4B
VAV1	vav guanine nucleotide exchange factor 1
ATP2B1	ATPase plasma membrane Ca2+ transporting 1
CD58	CD58 molecule
USP42	ubiquitin specific peptidase 42
ROCK1	Rho associated coiled-coil containing protein kinase 1
RNF13	ring finger protein 13
NADK	NAD kinase
STK10	serine/threonine kinase 10
CD46	CD46 molecule
CHD2	chromodomain helicase DNA binding protein 2
LSP1	lymphocyte-specific protein 1
CELF2	CUGBP Elav-like family member 2
MYO9B	myosin IXB
MX2	MX dynamin like GTPase 2

Symbol	Entrez Gene Name
TCF7L2	transcription factor 7 like 2
JAK3	Janus kinase 3
ATG9A	autophagy related 9A
ATP11A	ATPase phospholipid transporting 11A
NEK6	NIMA related kinase 6
PIAS1	protein inhibitor of activated STAT 1
KLF7	Kruppel like factor 7
GAB1	GRB2 associated binding protein 1
RAC2	ras-related C3 botulinum toxin substrate 2 (rho family, small GTP binding protein Rac2)
CBFA2T3	CBFA2/RUNX1 translocation partner 3
PCBP2	poly(rC) binding protein 2
TLE4	transducin like enhancer of split 4
AFF1	AF4/FMR2 family member 1
CYP4F12	cytochrome P450 family 4 subfamily F member 12
GNG7	G protein subunit gamma 7
PDLIM2	PDZ and LIM domain 2
RASSF5	Ras association domain family member 5
CASZ1	castor zinc finger 1
PTPRM	protein tyrosine phosphatase, receptor type M
CPA3	carboxypeptidase A3
THOP1	thimet oligopeptidase 1
EHMT2	euchromatic histone lysine methyltransferase 2
E2F6	E2F transcription factor 6
RORC	RAR related orphan receptor C
SNRPN	small nuclear ribonucleoprotein polypeptide N
NME6	NME/NM23 nucleoside diphosphate kinase 6
FUT10	fucosyltransferase 10
FLT3	fms related tyrosine kinase 3
CDK4	cyclin dependent kinase 4
PRKAR1B	protein kinase cAMP-dependent type I regulatory subunit beta
SAMD3	sterile alpha motif domain containing 3
PDE5A	phosphodiesterase 5A
VWF	von Willebrand factor
PRMT7	protein arginine methyltransferase 7
PDE3A	phosphodiesterase 3A
TRIM65	tripartite motif containing 65
FAH	fumarylacetoacetate hydrolase
ABCC3	ATP binding cassette subfamily C member 3
ZAP70	zeta chain of T-cell receptor associated protein kinase 70
CFH	complement factor H
SLC39A14	solute carrier family 39 member 14
DHODH	dihydroorotate dehydrogenase (quinone)
SPNS1	sphingolipid transporter 1 (putative)
MYLK	myosin light chain kinase
FCER1A	Fc fragment of IgE receptor Ia
ARHGAP6	Rho GTPase activating protein 6
SUSD3	sushi domain containing 3
DHRS3	dehydrogenase/reductase 3
BPNT1	3'(2'), 5'-bisphosphate nucleotidase 1
	MPL proto-oncogene, thrombopoietin receptor
SRC	SRC proto-oncogene, non-receptor tyrosine kinase
SLC8A3	solute carrier family 8 member A3
COL5A3	collagen type V alpha 3 chain
APBB3	amyloid beta precursor protein binding family B member 3
ARHGDIB	Rho GDP dissociation inhibitor beta
ARHGEF3	Rho guanine nucleotide exchange factor 3
BAD	BCL2 associated agonist of cell death
BCL2L1	BCL2 like 1
BIRC5	baculoviral IAP repeat containing 5
BLNK	B-cell linker
Bst2	bone marrow stromal cell antigen 2
C3	complement C3
CALCA	calcitonin related polypeptide alpha
CAMK2D	calcium/calmodulin dependent protein kinase II delta
CAMP	cathelicidin antimicrobial peptide
CAPG	capping actin protein, gelsolin like
CBFB	core-binding factor beta subunit
CCL4	C-C motif chemokine ligand 4

Symbol	Entrez Gene Name
Ccl9	chemokine (C-C motif) ligand 9
CCL17	C-C motif chemokine ligand 17
CCL20	C-C motif chemokine ligand 20
CCND1	cyclin D1
CCND2	cyclin D2
CCND3	cyclin D3
CCR9	C-C motif chemokine receptor 9
CD2	CD2 molecule
CD4	CD4 molecule
CD40	CD40 molecule
CD226	CD226 molecule
CD247	CD247 molecule
CDKN1B	cyclin dependent kinase inhibitor 1B
CDKN2A	cyclin dependent kinase inhibitor 2A
CEBPB	CCAAT/enhancer binding protein beta
COPS4	COP9 signalosome subunit 4
CR2	complement C3d receptor 2
CREM	cAMP responsive element modulator
CRKL	CRK like proto-oncogene, adaptor protein
CTNNB1	catenin beta 1
DLL1	delta like canonical Notch ligand 1
EGR2	early growth response 2
EHF	ETS homologous factor
EPO	erythropoietin
FABP5	fatty acid binding protein 5
FBXW7	F-box and WD repeat domain containing 7
FNIP1	folliculin interacting protein 1
FOXO3	forkhead box O3
FOXP3	forkhead box P3
FYB	FYN binding protein
GATA1	GATA binding protein 1
GATA2	GATA binding protein 2
GIMAP1-GIMAP5	GIMAP1-GIMAP5 readthrough
GP1BA	glycoprotein Ib platelet alpha subunit
GRAP2	GRB2-related adaptor protein 2
GRK2	G protein-coupled receptor kinase 2
GRK3	G protein-coupled receptor kinase 3
GZMB	granzyme B
HAVCR2	hepatitis A virus cellular receptor 2
HBB	hemoglobin subunit beta
HDAC1	histone deacetylase 1
HDAC2	histone deacetylase 2
HDAC11	histone deacetylase 11
HES1	hes family bHLH transcription factor 1
HIPK2	homeodomain interacting protein kinase 2
HLTF	helicase like transcription factor
HMOX1	heme oxygenase 1
ICAM1	intercellular adhesion molecule 1
ID2	inhibitor of DNA binding 2, HLH protein
IFI16	interferon gamma inducible protein 16
IFIT1B	interferon induced protein with tetratricopeptide repeats 1B
IFNB1	interferon beta 1
IFNG	interferon gamma
IGHM	immunoglobulin heavy constant mu
IKZF1	IKAROS family zinc finger 1
Il3	interleukin 3
IL4	interleukin 4
IL13	interleukin 13
IL15	interleukin 15
IL18	interleukin 18
IL10RA	interleukin 10 receptor subunit alpha
IL12RB1	interleukin 12 receptor subunit beta 1
IL4I1	interleukin 4 induced 1
IRF4	interferon regulatory factor 4
IRF6	interferon regulatory factor 6
IRF7	interferon regulatory factor 7
IRF8	interferon regulatory factor 8
ITGA2	integrin subunit alpha 2

Symbol	Entrez Gene Name
ITGA5	integrin subunit alpha 5
ITGA2B	integrin subunit alpha 2b
ITGB1	integrin subunit beta 1
ITGB2	integrin subunit beta 2
ITGB7	integrin subunit beta 7
ITK	IL2 inducible T-cell kinase
ITPR1	inositol 1,4,5-trisphosphate receptor type 1
JAK2	Janus kinase 2
KITLG	KIT ligand
KLF3	Kruppel like factor 3
KLRB1	killer cell lectin like receptor B1
LAT2	linker for activation of T-cells family member 2
LEP	leptin
LRP1	LDL receptor related protein 1
LRRC8A	leucine rich repeat containing 8 family member A
LTK	leukocyte receptor tyrosine kinase
Ly6a (includes others)	lymphocyte antigen 6 complex, locus A
MAP2K1	mitogen-activated protein kinase kinase 1
MAP3K8	mitogen-activated protein kinase kinase kinase 8
MEN1	menin 1
mevalonic acid	--
MKL1	megakaryoblastic leukemia (translocation) 1
MKL2	MKL1/myocardin like 2
MS4A1	membrane spanning 4-domains A1
MYC	MYC proto-oncogene, bHLH transcription factor
NCF2	neutrophil cytosolic factor 2
NCOR2	nuclear receptor corepressor 2
NCR2	natural cytotoxicity triggering receptor 2
NDRG1	N-myc downstream regulated 1
NDST2	N-deacetylase and N-sulfotransferase 2
NFKB1	nuclear factor kappa B subunit 1
NLRP3	NLR family pyrin domain containing 3
NTN1	netrin 1
PAG1	phosphoprotein membrane anchor with glycosphingolipid microdomains 1
PHB2	prohibitin 2
PIGA	phosphatidylinositol glycan anchor biosynthesis class A
PIK3CD	phosphatidylinositol-4,5-bisphosphate 3-kinase catalytic subunit delta
PIN1	peptidylprolyl cis/trans isomerase, NIMA-interacting 1
PIP5K1C	phosphatidylinositol-4-phosphate 5-kinase type 1 gamma
PLAU	plasminogen activator, urokinase
PLP1	proteolipid protein 1
POFUT1	protein O-fucosyltransferase 1
PPARG	peroxisome proliferator activated receptor gamma
PRDM1	PR/SET domain 1
PRKCB	protein kinase C beta
PRKCQ	protein kinase C theta
PSEN1	presenilin 1
PTK2B	protein tyrosine kinase 2 beta
PTPN11	protein tyrosine phosphatase, non-receptor type 11
PTPRCAP	protein tyrosine phosphatase, receptor type C associated protein
PXN	paxillin
RARA	retinoic acid receptor alpha
RCAN1	regulator of calcineurin 1
REL	REL proto-oncogene, NF-kB subunit
RGS10	regulator of G protein signaling 10
RGS13	regulator of G protein signaling 13
RHAG	Rh-associated glycoprotein
RSAD2	radical S-adenosyl methionine domain containing 2
SBDS	SBDS, ribosome maturation factor
SELE	selectin E
SELL	selectin L
SELP	selectin P
SELPLG	selectin P ligand
SKAP1	src kinase associated phosphoprotein 1
SLA	Src like adaptor
SLC2A1	solute carrier family 2 member 1
SMARCA4	SWI/SNF related, matrix associated, actin dependent regulator of chromatin, subfamily a, member 4
SOX4	SRY-box 4

Symbol	Entrez Gene Name
SOX5	SRY-box 5
SPHK2	sphingosine kinase 2
SPI1	Spi-1 proto-oncogene
SPIB	Spi-B transcription factor
SRF	serum response factor
STK4	serine/threonine kinase 4
TCF12	transcription factor 12
TFEC	transcription factor EC
TNFRSF11A	TNF receptor superfamily member 11a
TNFRSF1A	TNF receptor superfamily member 1A
TRAT1	T-cell receptor associated transmembrane adaptor 1
USF1	upstream transcription factor 1
USF2	upstream transcription factor 2, c-fos interacting
USP4	ubiquitin specific peptidase 4
USP18	ubiquitin specific peptidase 18
VCAM1	vascular cell adhesion molecule 1
YY1	YY1 transcription factor
ZEB2	zinc finger E-box binding homeobox 2
ZFPM1	zinc finger protein, FOG family member 1

Supplementary Table 4. Sequences of PCR primers and oligonucleotides.

Human genes are indicated by uppercase letters and mouse genes are indicated by lowercase.

Gene	Forward	Reverse
DRAIR	GACTGTAGGCTGAAACTGACC	TTTCCGTGTCTTCGCTACTG
CPEB2	CGAGTTGCTTTCTCCAATCAG	ACACATCTGGTCATCTAGCAC
NEAT1	TGGAGGAGTCAGGAGGAATAG	GGCATGGACAAGTTGAAGATTAG
PPIA	GACTGAGTGGTTGGATGGCAAG	CGCTTATTCCTGGACCCAAAGCG
IL1RN	CCTCATGCTCTGTTCTTGGG	TGTCCTGCTTTCTGTTCTCG
TNF	CCCTGAAAACAACCCTCAGA	GTCCTTTCCAGGGGAGAGAG
FCGR3B	GGTCACTGGGATTGCTGTAG	TGGGATTGGCTGGTTTCTC
CD36	GCCAGGTATTGCAGTTCTTTTC	TGTCTGGGTTTTCAACTGGAG
MCL1	AGGAGATGGAAGCCCCG	CAGATTCCCCGACCAACTC
KLF4	GGGTTTTGGGTTTTGGCTTC	GAACGTGGAGAAAGATGGGAG
IL1B	CAAAGGCGGCCAGGATATAA	CTAGGGATTGAGTCCACATTCAG
GAPDH	CTTTTGCGTCGCCAGCCGAG	CCAGGCGCCCAATACGACCA
HPRT1	GTATTCATTATAGTCAAGGGCATATCC	AGATGGTCAAGGTCGCAAG
Drair	TCCATTTGCCTAGGAACCAAG	GAGCTTCAGGTTACAGTCCATC
Cpeb2	CCAGAACTCAAATACCCAAAAGG	TGGTCATCTAGCACATATGGC
Il1rn	TCATAGTGTGTTCTTGGGCATC	AGCGGATGAAGGTAAAGCG
Il1b	ACGGACCCCAAAAGATGAAG	TTCTCCACAGCCACAATGAG
Tnf	TGTTGCCTCCTCTTTTGCTT	TGGTCACCAAATCAGCGTTA
Il6	GATGCTACCAAACTGGATATAATCAG	CTCTGAAGGACTCTGGCTTTG
Rplpo	TTATAACCCTGAAGTGCTCGAC	GCGCTTGTACCCATTGATG
**ChIP/ChIRP primers**		
DRAIR-pro	CACGGCGTAAGGACCAT	AAATGAGGGATTTCGGGTTTG
CPEB2-pro	TAGCCGAGGCTTCATGTTTAC	CGCCCTCTAAGCTTGTTTCT
IL1RN-pro	AGATGCAAGCCTTCAGGTAAG	TGATATGTTTCCAGTCTCCAGC
FCGR3B-pro	CTGTGTCGTGTCGGAATCTTAT	GAGGGTCAGCCTGATTGTATG
MCL1-pro	GATGGATGGGAACAACAGTCTTA	ACGTGGAGCATCCTCATTTC
OPTC-Dbs	CCATGTGCTCTCTCTCTTTCTC	CCTCTGTGCTCTCATCCTATTTC

**Human *DRAIR* biotinylated ChIRP Probes from Stellaris**	
TTGACAGAGCATGGTTGACA	
CAATGCTACAGTTATCCACA	
TTTGTCATCAGGGGAAGATT	
AGAAGTCAGTCAGTCTACGT	
CTCACTGTTTATGACTCTGC	
ATCTTGTTGAGCAATAGGGC	
ATAGGAACATGTCCACACGG	
AGGAGGATGAAGCTCTTGTT	
TTGACAGAGCATGGTTGACA	

REFERENCES

1. Chen EY, Tan CM, Kou Y, Duan Q, Wang Z, Meirelles GV, Clark NR, Ma'ayan A. Enrichr: interactive and collaborative HTML5 gene list enrichment analysis tool. BMC Bioinformatics. 2013;128(14).

2. Kuleshov MV, Jones MR, Rouillard AD, Fernandez NF, Duan Q, Wang Z, Koplev S, Jenkins SL, Jagodnik KM, Lachmann A, McDermott MG, Monteiro CD, Gundersen GW, Ma'ayan A. Enrichr: a comprehensive gene set enrichment analysis web server 2016 update. Nucleic Acids Research. 2016; gkw377 .

3. Yu-Jian Kang , De-Chang Yang , Lei Kong , Mei Hou , Yu-Qi Meng , Liping Wei , Ge Gao CPC2: A Fast and Accurate Coding Potential Calculator Based on Sequence Intrinsic Features. Nucleic Acids Res 2017 45:W12-W16.
